# Establishment of *w*Mel *Wolbachia* in *Aedes aegypti* mosquitoes and reduction of local dengue transmission in Cairns and surrounding locations in northern Queensland, Australia

**DOI:** 10.12688/gatesopenres.13061.2

**Published:** 2020-04-08

**Authors:** Peter A. Ryan, Andrew P. Turley, Geoff Wilson, Tim P. Hurst, Kate Retzki, Jack Brown-Kenyon, Lauren Hodgson, Nichola Kenny, Helen Cook, Brian L. Montgomery, Christopher J. Paton, Scott A. Ritchie, Ary A. Hoffmann, Nicholas P. Jewell, Stephanie K. Tanamas, Katherine L. Anders, Cameron P. Simmons, Scott L. O’Neill

**Affiliations:** 1 Institute of Vector-Borne Disease, Monash University, Clayton, Victoria, 3800, Australia; 2 Biosecurity and Agricultural Services, Department of Jobs, Precincts and Regions, Victoria State Government, Atwood, Victoria, Australia; 3 Metro South Public Health Unit, Queensland Health, Coopers Plains, Queensland, Australia; 4 College of Public Health, Medical and Veterinary Sciences, James Cook University, Cairns, Queensland, Australia; 5 School of Biosciences, Bio21 Institute, University of Melbourne, Parkville, Victoria, Australia; 6 Division of Epidemiology and Biostatistics, School of Public Health, University of California, Berkeley, California, USA; 7 Centre for Statistical Methodology, London School of Hygiene and Tropical Medicine, London, UK; 8 Oxford University Clinical Research Unit, Hospital for Tropical Diseases, Ho Chi Minh City, Vietnam

**Keywords:** Dengue, World Mosquito Program, Eliminate Dengue, Wolbachia, Aedes aegypti, mosquito release, community engagement

## Abstract

**Background: **The *w*Mel strain of *Wolbachia* has been successfully introduced into *Aedes aegypti* mosquitoes and subsequently shown in laboratory studies to reduce transmission of a range of viruses including dengue, Zika, chikungunya, yellow fever, and Mayaro viruses that cause human disease. Here we report the entomological and epidemiological outcomes of staged deployment of *Wolbachia* across nearly all significant dengue transmission risk areas in Australia.

**Methods: **The *w*Mel strain of *Wolbachia* was backcrossed into the local *Aedes aegypti* genotype (Cairns and Townsville backgrounds) and mosquitoes were released in the field by staff or via community assisted methods. Mosquito monitoring was undertaken and mosquitoes were screened for the presence of *Wolbachia*. Dengue case notifications were used to track dengue incidence in each location before and after releases.

**Results: **Empirical analyses of the *Wolbachia* mosquito releases, including data on the density, frequency and duration of *Wolbachia* mosquito releases, indicate that *Wolbachia* can be readily established in local mosquito populations, using a variety of deployment options and over short release durations (mean release period 11 weeks, range 2-22 weeks). Importantly, *Wolbachia* frequencies have remained stable in mosquito populations since releases for up to 8 years. Analysis of dengue case notifications data demonstrates near-elimination of local dengue transmission for the past five years in locations where *Wolbachia* has been established. The regression model estimate of *Wolbachia *intervention effect from interrupted time series analyses of case notifications data prior to and after releases, indicated a 96% reduction in dengue incidence in *Wolbachia *treated populations (95% confidence interval: 84 – 99%).

**Conclusion: **Deployment of the *w*Mel strain of *Wolbachia* into local *Ae. aegypti* populations across the Australian regional cities of Cairns and most smaller regional communities with a past history of dengue has resulted in the reduction of local dengue transmission across all deployment areas.

## Introduction

The *w*Mel strain of *Wolbachia* has been successfully introduced into *Aedes aegypti* mosquitoes and subsequently shown in laboratory studies to reduce transmission of a range of viruses including dengue, Zika, chikungunya, yellow fever, and Mayaro viruses that cause human disease ( Aliota *et al.*, 2016a; Aliota *et al.*, 2016b; Amuzu *et al.*, 2015; Caragata *et al.*, 2016; Caragata *et al.*, 2019; Carrington *et al.*, 2018; Dutra *et al.*, 2016; Ferguson *et al.*, 2015; Frentiu *et al.*, 2014; Kho *et al.*, 2016; Moreira *et al.*, 2009; Pereira *et al.*, 2018; Rocha *et al.*, 2019; Tan *et al.*, 2017; van den Hurk *et al.*, 2012; Walker *et al.*, 2011; Ye *et al.*, 2013; Ye *et al.*, 2015; Ye *et al.*, 2016) Early field trials involving releases of *Wolbachia* infected *Ae. aegypti* mosquitoes into two isolated communities in northern Australia showed that the *w*Mel strain of *Wolbachia* could be deployed and establish in the local mosquito populations with full community support ( Hoffmann *et al.*, 2011) and persist ( Hoffmann *et al.*, 2014). Further it was shown that the dengue blocking properties of these mosquitoes remained stable several years after establishment ( Frentiu *et al.*, 2014). Additional releases into a number of urban settings in Cairns in northern Australia subsequently assessed the effects of release area size and landscape features on *Wolbachia* establishment and spread into the mosquito populations ( Schmidt *et al.*, 2017). Subsequent city-wide *Wolbachia* mosquito releases were undertaken across the medium-sized city of Townsville in northern Queensland, resulting in successful establishment of *Wolbachia* in local mosquito populations and complete elimination of local dengue transmission ( O’Neill *et al.*, 2018).

All prior field studies resulted in a patchwork of deployments of various sizes across areas of north Queensland, Australia ( Hoffmann *et al.*, 2011; Hoffmann *et al.*, 2014; O’Neill *et al.*, 2018; Schmidt *et al.*, 2017). From 2013 to 2017 the World Mosquito Program (formerly known as the Eliminate Dengue Program) undertook a series of additional deployments in nearly all significant dengue transmission risk areas in Australia where *w*Mel *Wolbachia* had not yet been deployed. These releases involved a variety of release methods, including releases of eggs and adult stages directly by project staff and through community assisted methods involving school children, businesses, community groups, and individual householders. Here we describe the entomological and epidemiological outcomes of this work.

## Methods

### Intervention area

**Figure 1.**
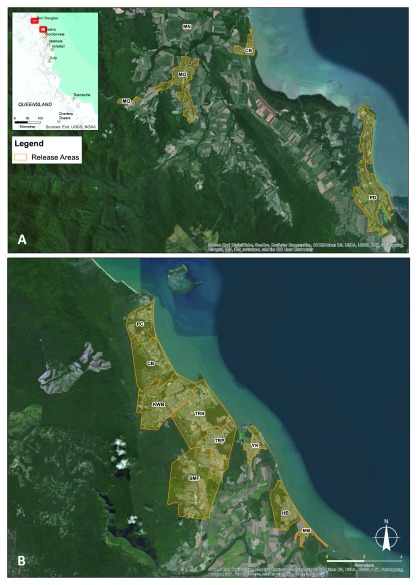
Map of Douglas release areas. Cooya Beach (CB), Mossman (MO), Mossman Gorge (MG), Mossman North (MN), Port Douglas (PD) ( **A**) and Cairns (northern) release areas: Clifton Beach (CB), Holloways Beach (HB), Kewarra Beach (KWB), Machans Beach (MB), Palm Cove (PC), Trinity Beach (TRB), Trinity Park (TRP), Smithfield (SMF), Yorkeys Knob (YK) ( **B**).

**Figure 2.**
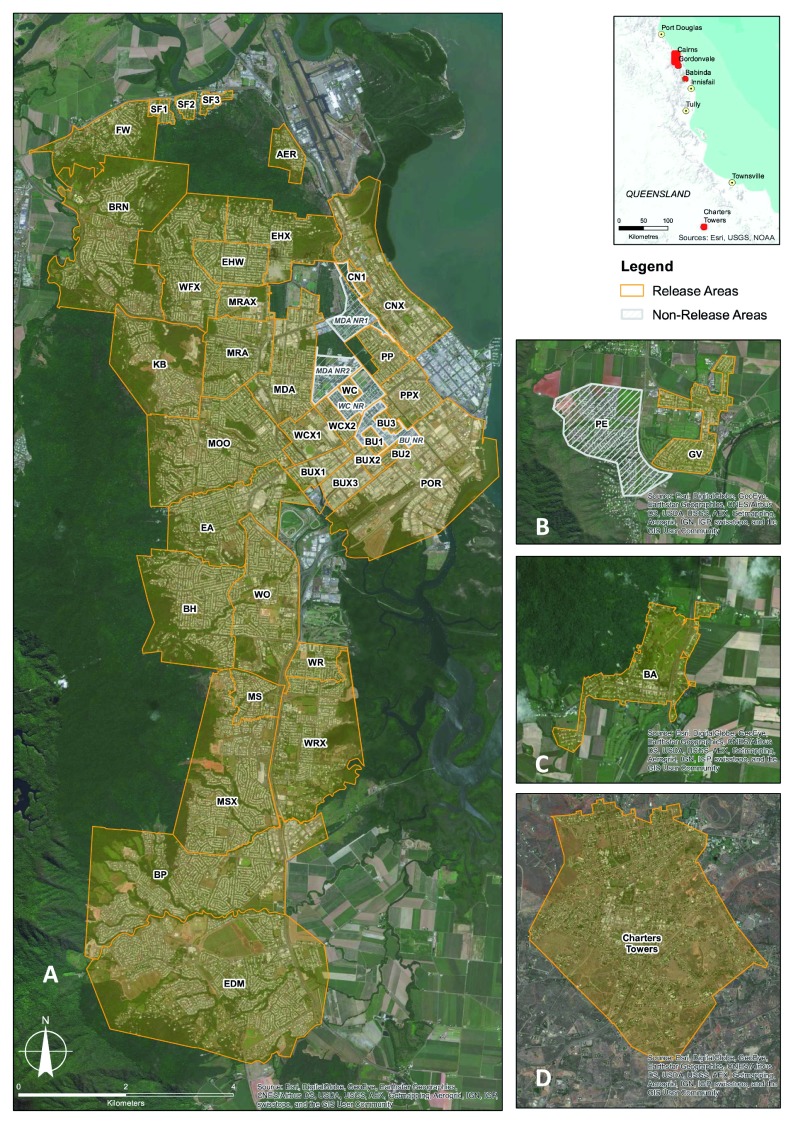
Map of Cairns (central) release areas and non-release areas. Aeroglen (AER), Bayview Heights (BH), Bentley Park (BP), Brinsmead (BRN), Bungalow 1 (BU1), Bungalow 2 (BU2), Bungalow 3 (BU3), Bungalow Ext 1 (BUX1), Bungalow Ext 2 (BUX2), Bungalow Ext 3 (BUX3), Bungalow non-release area (BU NR), Cairns North 1 (CN1), Cairns North Ext (CNX), Earlville (EA), Edge Hill Ext (EHX), Edge Hill/Whitfield (EHW), Edmonton (EDM), Freshwater (FW), Kanimbla (KB), Manoora (MRA), Manoora Ext (MRAX), Manunda (MDA), Manunda non-release area 1 (MDA NR1), Manunda non-release area 2 (MDA NR2), Mooroobool (MOO), Mount Sheridan (MS), Mount Sheridan Ext (MSX), Parramatta Park (PP), Parramatta Park Ext (PPX), Portsmith (POR), Stratford 1 (SF1), Stratford 2 (SF2), Stratford 3 (SF3), Westcourt (WC), Westcourt non-release area (WC NR), Westcourt Ext 1 (WCX1), Westcourt Ext 2 (WCX2), White Rock (WR), White Rock Ext (WRX), Whitfield Ext (WFX), Woree (WO) ( **A**), Gordonvale (GV) release area and Pyramid Estate non-release area (PE) ( **B**); Babinda (BA) release area ( **C**), and Charters Towers (CT) release area ( **D**).

**Figure 3.**
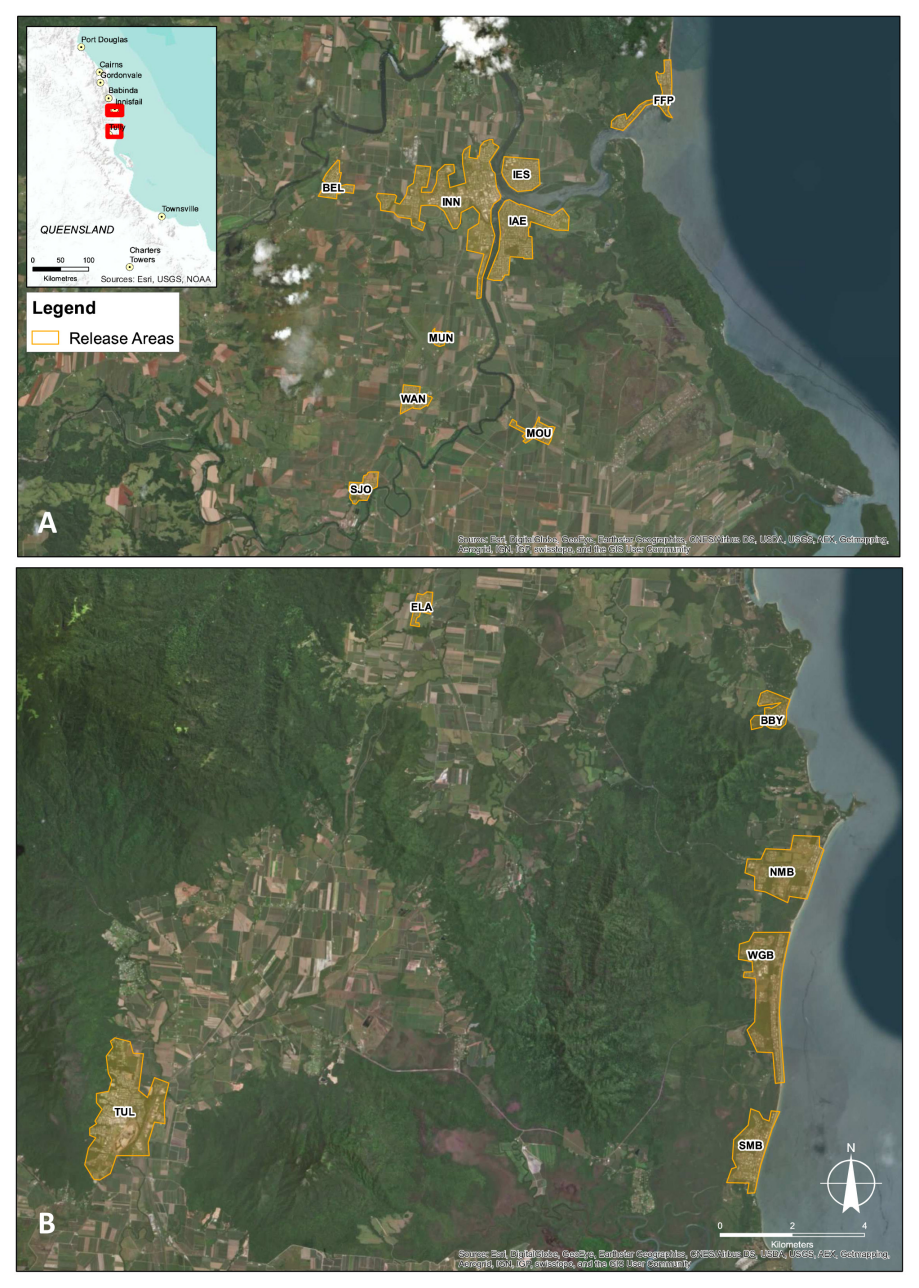
Map of Cassowary Coast - Innisfail release areas. Belvedere (BEL), Innisfail (INN), Innisfail Estate (IES), Flying Fish Point (FFP), Innisfail East (IAE), Mundoo (MUN), Wagan (WAN), Mourilyan (MOU), South Johnstone (SJO) ( **A**) and Cassowary Coast – Tully release areas Bingal Bay (BBY), El Arish (ELA), North Mission Beach (NMB), Wongaling Beach (WGB), South Mission Beach (SMB), Tully (TUL) ( **B**).

*Wolbachia* mosquito releases were undertaken across four local government administrative areas in northern Queensland, Australia: the Cairns Region, the Cassowary Coast Region, the Douglas Shire and the Charters Towers Region ( Table 1, Figure 1– Figure 3). Within each region, the locations for *Wolbachia* mosquito releases were selected based on historical dengue case reports, human population density, reported presence of *Ae. aegypti* and logistical considerations. Depending on the specific objective of each *Wolbachia* release, for example small scale releases to test different release methods (e.g. egg release trials in Stratford 1–3, Cairns North, Bungalow 1–3, Table 1, Figure 2), or an area-wide release across the entire location (e.g. adult releases across 23 Cairns suburbs between Nov 2016 and Jun 2017, Table 1, Figure 1B, Figure 2), each area was mapped and the target release areas (generally the residential areas and some business areas) were identified. Areas deemed unsuitable for *Ae. aegypti*, such as uninhabited forested and vegetated areas, open or vacant areas, sporting fields, large industrial and commercial areas, agricultural and farming areas, and major transport infrastructure (major roads, highways, railways and airports) were excluded from releases. The bounded size (km ^2^) of each release area was calculated, along with the residential population and the number of households ( Table 1).

**Table 1.** Summary Information for release sites.

Region/Shire	Release Area name (code)	Size (km ^2^)	Population	Number of houses	Release type (E = eggs, A = Adults)	Release start date mm/yyyy)	Release frequency (1 = weekly, 2 = every 2 weeks)	Release duration (weeks)	Release Description
Cairns	Gordonvale (GV) *	1.15	1904	796	A	01/2011	1	10	Weekly releases at 1:3 houses, release methods and monitoring results to October 2014 described in Hoffmann *et al.*, 2011, Hoffmann *et al.*, 2014.
	Yorkeys Knob (YK) *	0.78	2196	1,218	A	01/2011	1	10	Weekly releases at 1:3 houses, release methods and monitoring results to October 2014 described in Hoffmann *et al.*, 2011, Hoffmann *et al.*, 2014.
	Edge Hill/Whitfield (EHW)	0.94	2,251	975	A	01/2013	1	15	Release at 1:5 households, 70 adult mosquitoes per release point, release methods and monitoring results to April 2015 described in Schmidt *et al.*, 2017.
	Parramatta Park (PP)	0.49	2,167	1,173	A	01/2013	1	15	Release at 1:5 households, 70 adult mosquitoes per release point, release methods and monitoring results to April 2015 described in Schmidt *et al.*, 2017.
	Westcourt (WC)	0.10	250	112	A	01/2013	1	16	Release at 1:5 households, 70 adult mosquitoes per release point, release methods and monitoring results to April 2015 described in Schmidt *et al.*, 2017.
	Babinda (BA) *	1.18	900	476	E+A	07/2013	1	9	Weekly releases of adults (10 weeks) or eggs (9 weeks) at 1:9 houses (82% adult releases and 18% egg releases), 25 adult mosquitoes per cup, 150 eggs per container (target emergence of 100 adults per container)
	Machans Beach (MB)	0.39	1,039	453	E+A	07/2013	1	11	Weekly releases of adults (11 weeks) or eggs (10 weeks) at 1:6 houses (70% adult releases and 30% egg releases), 25 adult mosquitoes per release cup, 150 eggs per container (target emergence of 100 adults per container)
	Stratford 1 (SF1)	0.17	354	155	E	6/2014	1	5	Release at 1:5 households, 75 viable eggs per container
	Stratford 2 (SF2)	0.15	291	114	E	6/2014	1	16	Release at 1:5 households, 75 viable eggs per container
	Stratford 3 (SF3)	0.14	221	113	E	6/2014	1	23	Release at 1:5 households, 75 viable eggs per container
	Bungalow 1 (BU1)	0.10	250	130	E	07/2014	1	13	Release at 1:10 households, 75 viable eggs per container
	Bungalow 2 (BU2)	0.12	358	232	E	07/2014 09/2015	1 2	9 16	2014 - Release at 1:5 households 75 eggs per container; 2015 - Release at 1:5 households, 75 viable eggs per container
	Bungalow 3 (BU3)	0.11	331	205	E	07/2014	1	13	Release at 1:10 households, 75 viable eggs per container
	Cairns North (CN1)	0.12	435	205	E	08/2014	1	13	Release at 1:5 households, 75 viable eggs per container
	Manunda (MDA)	1.31	3,860	1,810	E	05/2015	2	14	Release at 1:5 households, week release period, 100 viable eggs per container
	Manoora (MRA)	1.35	4,189	1,952	E	06/2015	2	11	Release at 1:5 households, 100 viable eggs per container
	Mooroobool (MOO)	3.00	7,208	2,883	E	06/2015	2	15	Release at 1:5 households, 100 viable eggs per container
	Earlville (EA)	1.81	4,031	2,126	E	07/2015	2	13	Release at 1:5 households, 100 viable eggs per container
	Woree (WO)	1.80	4,842	2,454	E	08/2015	2	10	Release at 1:5 households, 100 viable eggs per container
	Bungalow Ext 1 (BUX1)	0.30	432	222	E	09/2015	2	12	Release at 1:5 households, 100 viable eggs per container
	Westcourt Ext 1 (WCX1)	0.42	1,471	626	E	09/2015	2	13	Release at 1:5 households, 100 viable eggs per container
	Mount Sheridan (MS)	0.50	1,437	615	E	09/2015	2	6	Release at 1:5 households, week release period, 100 viable eggs per container
	White Rock (WR)	0.55	1,380	672	E	09/2015	2	11	Release at 1:5 households, 100 viable eggs per container
	Bungalow Ext 2 (BUX2)	0.22	218	127	E	10/2015	2	12	Release at 1:5 households, 100 viable eggs per container
	Westcourt Ext 2 (WCX2)	0.25	609	336	E	10/2015	2	12	Release at 1:5 households, 100 viable eggs per container
	Bentley Park (BP)	3.50	8,009	2,796	A	11/2016	1	12	Releases across 100 × 100 m grid, 100 *w*Mel adult mosquitoes per grid per week, egg releases undertaken by school children
	Edmonton (EDM)	6.08	10,696	4,049	A	11/2016	1	12	Releases across 100 × 100 m grid, 100 *w*Mel adult mosquitoes per grid per week
	Bayview Heights (BH)	2.38	4,245	1,644	A	02/2017	1	10	Releases across 100 × 100 m grid, 100 *w*Mel adult mosquitoes per grid per week
	Kanimbla (KB)	1.35	2,654	939	A	02/2017	1	11	Releases across 100 × 100 m grid, 100 *w*Mel adult mosquitoes per grid per week
	Mount Sheridan Ext (MSX)	2.88	6,811	2,520	A	02/2017	1	10	Releases across 100 × 100 m grid, 100 *w*Mel adult mosquitoes per grid per week
	White Rock Ext (WRX)	2.32	3,345	1,262	A	02/2017	1	12	Releases across 100 × 100 m grid, 100 *w*Mel adult mosquitoes per grid per week
	Brinsmead (BRN)	2.99	5,369	2,121	A	03/2017	1	10	Releases across 100 × 100 m grid, 100 *w*Mel adult mosquitoes per grid per week
	Cairns North Ext (CNX)	1.07	5,397	2,899	A	03/2017	1	2	Releases across 100 × 100 m grid, 100 *w*Mel adult mosquitoes per grid per week
	Edge Hill Ext (EHX)	1.14	2,366	1,174	A	03/2017	1	10	Releases across 100 × 100 m grid, 100 *w*Mel adult mosquitoes per grid per week
	Manoora Ext (MRAX)	0.33	1,827	1,018	A	03/2017	1	10	Releases across 100 × 100 m grid, 100 *w*Mel adult mosquitoes per grid per week
	Parramatta Park Ext (PPX)	0.28	1,185	466	A	03/2017	1	10	Releases across 100 × 100 m grid, 100 *w*Mel adult mosquitoes per grid per week
	Portsmith (POR)	2.82	256	50	A	03/2017	1	10	Releases across 100 × 100 m grid, 100 *w*Mel adult mosquitoes per grid per week
	Whitfield Ext (WFX)	1.83	3,579	1564	A	03/2017	1	10	Releases across 100 × 100 m grid, 100 *w*Mel adult mosquitoes per grid per week
	Bungalow Ext 3 (BUX3)	0.50	13	7	A	04/2017	1	10	Releases across 100 × 100 m grid, 100 *w*Mel adult mosquitoes per grid per week
	Aeroglen (AER)	0.24	393	160	A	05/2017	1	10	Releases across 100 × 100 m grid, 100 *w*Mel adult mosquitoes per grid per week
	Holloways Beach (HB)	1.06	2,330	1,187	A	05/2017	1	10	Releases across 100 × 100 m grid, 100 *w*Mel adult mosquitoes per grid per week
	Kewarra Beach (KWB)	3.31	5,670	2,370	A	05/2017	1	10	Releases across 100 × 100 m grid, 100 *w*Mel adult mosquitoes per grid per week
	Smithfield (SMF)	3.13	5,178	2,131	A	05/2017	1	10	Releases across 100 × 100 m grid, 100 *w*Mel adult mosquitoes per grid per week
	Trinity Beach (TRB)	3.22	5,429	2,558	A	05/2017	1	10	Releases across 100 × 100 m grid, 100 *w*Mel adult mosquitoes per grid per week
	Clifton Beach (CB)	2.57	3,135	1,536	A	06/2017	1	10	Releases across 100 × 100 m grid, 100 *w*Mel adult mosquitoes per grid per week
	Freshwater (FW)	1.16	2,014	962	A	06/2017	1	10	Releases across 100 × 100 m grid, 100 *w*Mel adult mosquitoes per grid per week
	Palm Cove (PC)	1.03	1,791	1,213	A	06/2017	1	10	Releases across 100 × 100 m grid, 100 *w*Mel adult mosquitoes per grid per week
	Trinity Park (TRP)	1.25	3,098	1,273	A	06/2017	1	10	Releases across 100 × 100 m grid, 100 *w*Mel adult mosquitoes per grid per week
	Pyramid Estate (PE)	1.99	3,153	1135		None	None	None	No releases
	Bungalow non-release area (BUN NR)	0.22	570	307		None	None	None	No releases
	Manunda non-release area 1 (MDA NR1)	0.53	1,288	807		None	None	None	No releases
	Manunda non-release area 2 (MDA NR2)	0.13	310	143		None	None	None	No releases
	Westcourt non release area (WC NR)	0.36	1,532	869		None	None	None	No releases
Cassowary Coast - Innisfail	Belvedere (BEL)	0.43	885	351	A	03/2017	1	14	Releases across 100 × 100 m grid, 100 *w*Mel adult mosquitoes per grid per week
	Flying Fish Point (FFP)	0.43	635	344	A	03/2017	1	16	Releases across 100 × 100 m grid, 100 *w*Mel adult mosquitoes per grid per week
	Innisfail East (IAE)	1.77	2,665	1,241	A	03/2017	1	14	Releases across 100 × 100 m grid, 100 *w*Mel adult mosquitoes per grid per week
	Innisfail Estate (IES)	0.63	1,333	624	A	03/2017	1	14	Releases across 100 × 100 m grid, 100 *w*Mel adult mosquitoes per grid per week
	Mundoo (MUN)	0.13	162	66	A	28/02/2017	1	16	Releases across 100 × 100 m grid, 100 *w*Mel adult mosquitoes per grid per week
	Wangan (WAN)	0.33	590	256	A	02/2017	1	16	Weekly releases across 100 × 100 m grid, 100 *w*Mel adult mosquitoes per grid per week
	Innisfail (INN)	1.95	3,331	1,439	A	03/2017	1	14	Releases across 100 × 100 m grid, 100 *w*Mel adult mosquitoes per grid per week, *w*AlbB infected male- only *Ae. aegypti* mosquito releases were undertaken for 26 weeks between Dec 2017 and Jun 2018
	Mourilyan (MOU)	0.29	420	195	A	02/2017	1	16	Releases across 100 × 100 m grid, 100 *w*Mel adult mosquitoes per grid per week, *w*AlbB infected male- only *Ae. aegypti* mosquito releases were undertaken for 28 weeks between Nov 2017 and Jun 2018
	South Johnstone (SJO)	0.34	390	175	A	28/02/2017	1	16	Releases across 100 × 100 m grid, 100 *w*Mel adult mosquitoes per grid per week, *w*AlbB infected male- only *Ae. aegypti* mosquito releases were undertaken for 26 weeks between Dec 2017 and Jun 2018
Cassowary Coast - Tully	Bingal Bay (BBY)	0.48	348	191	A	05/2017	1	12	Releases across 100 × 100 m grid, 100 *w*Mel adult mosquitoes per grid per week
	El Arish (ELA)	0.24	250	137	A	05/2017	1	12	Releases across 100 × 100 m grid, 100 *w*Mel adult mosquitoes per grid per week
	North Mission Beach (NMB)	0.99	639	552	E+A	05/2017	1	12	Releases across 100 × 100 m grid, 100 *w*Mel adult mosquitoes per grid per week, egg releases undertaken by school children
	South Mission Beach (SMB)	1.03	931	547	A	05/2017	1	12	Releases across 100 × 100 m grid, 100 *w*Mel adult mosquitoes per grid per week
	Tully (TUL)	1.74	2,215	976	A	05/2017	1	12	Releases across 100 × 100 m grid, 100 *w*Mel adult mosquitoes per grid per week
	Wongaling Beach (WGB)	1.4	1,181	846	A	05/2017	1	12	Releases across 100 × 100 m grid, 100 *w*Mel adult mosquitoes per grid per week
Charters Towers	Charters Towers (CT)	6.93	6,996	3,359	E+A	10/2016	1	8	Weekly releases across 100 × 100 m grid, 100 *w*Mel adult mosquitoes per grid per week, egg releases undertaken by school children and community volunteers
Douglas	Cooya Beach (CB)	0.45	888	395	E+A	10/2016	1	8	Weekly releases across 100 × 100 m grid, 100 *w*Mel adult mosquitoes per grid per week, egg releases undertaken by community volunteers
	Mossman (MO)	1.90	1,862	927	E+A	10/2016	1	8	Weekly releases across 100 × 100 m grid, 100 *w*Mel adult mosquitoes per grid per week, egg releases undertaken by community volunteers
	Mossman Gorge (MG)	0.10	125	49	E+A	10/2016	1	7	Weekly releases across 100 × 100 m grid, 100 *w*Mel adult mosquitoes per grid per week, egg releases undertaken by community volunteers
	Mossman North (MN)	0.06	88	42	E+A	10/2016	1	8	Weekly releases across 100 × 100 m grid, 100 *w*Mel adult mosquitoes per grid per week, egg releases undertaken by community volunteers
	Port Douglas (PD)	4.59	4,318	2,585	E+A	10/2016	1	8	Weekly releases across 100 × 100 m grid, 100 *w*Mel adult mosquitoes per grid per week, egg releases undertaken by community volunteers

* In Gordonvale, Yorkeys Knob and Babinda, monitoring extended beyond the boundaries of the release area and demonstrated *Wolbachia* establishment throughout this extended area. The population denominator used for epidemiological analysis was therefore calculated for the larger monitored area as follows: Gordonvale, population 2136 in 1.31 km ^2^; Yorkeys Knob, population 2701 in 0.97 km ^2^; Babinda, population 1079 in 1.04 km ^2^.

### Community engagement

For *Wolbachia* mosquito releases between 2011–2014, communication and community engagement activities followed the approach described in Hoffmann *et al.* (2011). This included consultation with key stakeholders and community groups, one-on-one meetings, displays at community events and centers, and door-knocking and mail-outs to householders to assess support for and participation in *Wolbachia* mosquito releases. Prior to releases residents were asked to provide permission for release of *Wolbachia* mosquitoes, either as adult stages from the footpath near their house, or as eggs that were placed into containers by program staff in outdoor shaded areas at their properties. During the release period, we continued to undertake close engagement with the local communities to ensure that residents were informed about the study and were comfortable with continuation of release activities. This included random household surveys, meetings with reference groups of residents and community leaders, and ongoing promotion of a free phone number and accessible city project office in Cairns. Periodic result updates were provided to the communities through letterbox leaflet drops, attendance at community events and meetings, paid advertisements in local newspapers, community newsletters and radio and television media outlets. Residences of a small number of people that did not wish to participate were excluded from release activities.

For *Wolbachia* mosquito releases between 2015–2017, community engagement activities followed the Public Acceptance Model (PAM) as described in O’Neill *et al.* (2018). The PAM was comprised of the following four components:

1. Raising broad community and stakeholder awareness. Information was provided to residents and key stakeholders about *Wolbachia* and mosquito release and monitoring activities via various channels, including mass communication (newspapers, media events), school outreach programs and social media, and direct engagement through face-to-face meetings, stalls at community events and presentations at existing community networks and meetings, information kiosks and traditional electronic mails outs of information letters and updates.
2. Quantitative surveys to assess community awareness and support. Pre-release surveys were conducted by an external market research company (Compass Research) (see methods in O’Neill *et al.*, 2018), and were undertaken from July 2016 ( Table 2). In Charters Towers, Douglas and Cassowary Coast shires additional baseline surveys were undertaken prior to commencement of communication and engagement campaigns, along with an initial survey in Cairns in Nov 2013. Each survey involved 100–300 participants ( Table 2).
3. Establishment of an issues management system. The system enabled community members to easily contact the program with any questions and concerns and have them quickly addressed by program staff typically within 24 hours of receipt. The system also allowed residents to opt in or out of direct participation in release and monitoring activities.
4. Community reference group. Community reference groups were established in each location, with respected community members from key stakeholder groups ( Table 2). The reference group’s function was to independently review activities to ensure that engagement was carried out in accordance with our stated Public Participation Principles ( O’Neill *et al.*, 2018).

**Table 2.** Community Reference Group membership and results of telephone surveys seeking to understand community awareness and support for *Wolbachia* mosquito releases.

		Cairns		Charters Towers		Douglas Shire		Cassowary Coast	
Community Reference Group	No. of members	9		9		7		11	
	Membership representation	Local government, health, education, women’s advocacy, local business, tourism, social development, general community		Local government, environment, local business, arts and culture, senior citizens, general community		Local government, tourism, environment, health, Indigenous Australians, local business, education.		Local government, social development, local business, environment, agriculture, health, Indigenous Australians, women’s advocacy, tourism, general community	
			Pre-release survey	Baseline survey	Pre-release survey	Baseline survey	Pre-release survey	Baseline survey	Pre-release survey
Qualitative Survey	Sample size	300	200	200	200	200	200	200	100
	Date	11/2013	07/2016	07/2016	09/2016	08/2016	09/2016	12/2016	02/2017
	Awareness (unprompted)	21%	56%	31%	31%	29%	43%	32%	44%
	Awareness (prompted)	N/A	81%	55%	37%	55%	58%	61%	57%
	Heard about WMP (TV, radio, Newspaper)	45%	58%	73%	90%	83%	86%	84%	86%
	Comfortable or very comfortable with release	86%	85%	82%	88%	84%	87%	91%	89%
	Support	N/A	N/A	N/A	93%	N/A	92%	N/A	90%

In Cairns, the implementation of the PAM commenced in 2015 and included engagement of traditional mass media, establishment of the Cairns sign-up website (where residents registered their interest in participating in the project), leveraging of existing networks to spread information (particularly through educational institutions), direct-to-premise informative mail-outs, information kiosks at community events and public locations, engagement of high-level stakeholders, such as government representatives, indigenous interest groups and environmental advocacy groups, establishment and maintenance of independent community reference groups, maintenance of community feedback channels (telephone survey and online forms), and distribution of quarterly field trial updates to participants and stakeholders. In Charters Towers, Douglas Shire and the Cassowary Coast the PAM was implemented prior to releases.

### Rearing

***Release colony maintenance.*** Two *Ae. aegypti w*Mel-infected lines were used in releases. The Cairns *Ae. aegypti w*Mel-infected line was released across Cairns, Douglas Shire and the Cassowary Coast areas, and a Townsville *Ae. aegypti w*Mel-infected line was released in Charters Towers, based on the proximity of the latter to Townsville where releases were undertaken between 2014–2015 (see description of the Townsville *Ae. aegypti w*Mel-infected line in O’Neill *et al.*, 2018). The Cairns *Ae. aegypti w*Mel-infected line (described in Walker *et al.*, 2011) was backcrossed to the offspring of Cairns field collected mosquitoes for six generations ( Hoffmann *et al.*, 2011). Between 2011 and 2015 the *w*Mel-infected mosquito colony was maintained at James Cook University, Cairns, in large semi-field cages ( Ritchie *et al.*, 2011) using methods described previously ( Hoffmann *et al.*, 2011). Cages contained up to 10,000 *w*Mel-infected *Ae. aegypti* and were provided access to human volunteer blood-feeders almost daily. To minimize laboratory adaptation, male *Ae. aegypti* pupae/adults from field collected *Ae. aegypti* (F1–F2 eggs) were introduced into the colony each generation, so that they constituted around 10–20% of the new male population. Eggs were collected on flannel cloth in plastic buckets acting as oviposition sites that were spread throughout the cage, incubated for 3 days and then stored in the laboratory.

From 2016 the *w*Mel-infected mosquito colony was maintained at Monash University, Melbourne. The Townsville *Ae. aegypti w*Mel-infected line was established by backcrossing the Cairns *Ae. aegypti w*Mel-infected line to the offspring of Townsville collected wildtype mosquitoes for three generations ( O’Neill *et al.*, 2018). Both *w*Mel-infected lines were maintained in controlled laboratory conditions, in 30 cm ^2^ mesh-sided rearing cages (see description of methods in O’Neill *et al.*, 2018). Each cage contained ~600 adults, and was fed using human volunteers once per week for two gonotrophic cycles. These colonies comprised of a broodstock, and a release-production colony. Male *Ae. aegypti* adults (from F1–F3 field collected material) were introduced into the broodstock cages at a rate of 10% every generation. Material from the broodstock colony was then transferred to the release-production colony where material was amplified through one generation without the addition of field collected males. Eggs were collected on red flannel cotton strips, and were matured for four days before being dried. Once the drying process was complete ( O’Neill *et al.*, 2018), eggs were packed and shipped to field sites.

***Adult mosquito rearing for releases.*** Between 2011–2013, adult mosquitoes for releases were produced using previously described methods ( Hoffmann *et al.*, 2011). Briefly, immature stages were reared in 3 L buckets with 2 L of water and fed a diet of Tetramin Tropical Tablets (Tetra Holding [US] Inc. Germany, Product number 16110) (2011: 150 larvae per bucket) or ground Tetramin Tropical Flakes (Tetra Holding [US] Inc. Germany, Product number 77101) (2012–2013: 500 larvae/bucket). When approximately 90% of larvae had pupated, the larvae/pupae were sieved and the required number of larvae/pupae were then separated into individual 750 mL plastic containers with approximately 200 ml of water. Adults were allowed to emerge and were maintained for 4–6 days on a 50% honey solution. The cups were then stacked into polystyrene boxes for transport to the release site for release.

For the 2016 Charters Towers releases, *w*Mel-infected mosquito eggs were produced at Monash University and were then shipped to Townsville where the eggs were hatched and the immatures stages were reared to adults in cups in a laboratory maintained at 25–28 °C. Egg strips were either hatched in tap water containing Aqua One Vege Wafers (Aqua Pacific UK Ltd, Southampton, UK, Product number 26050), and two days later approximately 100 larvae were aliquoted into individual release cups (paper drinking cups, 550 mL volume, 100 mm width × 180 mm height, C-DC9787, FPA, Australia) each containing 360 mL of tap water, or egg strips were cut into sections based on the required number of eggs to produce a target hatch of 100 larvae and the eggs were added directly to the individual rearing cups. Immatures were fed Aqua One Vege Wafers (1.5 wafers upon setup and 1.5 wafers on day 4). A mesh cover was placed on each cup and adults were maintained for 4–6 days on a 50% honey solution. Release cups were transferred to plastic tubs and transported to Charters Towers for release the following day between 0800–1000 hours. For the 2016–2017 Cairns releases, adult mosquitoes were reared as above, with initial rearing being undertaken at the James Cook University insectary and this transitioned to a laboratory in Cairns, maintained at 25–28 °C, in early 2017.

For the 2017 Douglas Shire releases, *w*Mel-infected mosquito eggs were produced at Monash University and were then shipped to Port Douglas where the eggs were hatched and the immatures stages were reared to adults in a laboratory maintained at 25–28 °C. Rearing followed above procedures except that all eggs were hatched in water containing 1 Tetramin Tropical Tablet, and two days later approximately 100 larvae were aliquoted into individual release cups (Plastic [PET] drinking cup, 425 mL volume, Detpak, Australia) each containing 300 mL of tap water. Plastic (PET) lids (Detpak, Australia) were placed on top of a mesh cover on each cup and adults were maintained for 4–6 days on 50% honey solution. On the morning of release, release cups were transferred to plastic tubs and transported to the field for release between 0800–1000 hours.

### Adult mosquito releases

Between 2011 and 2015, weekly releases were undertaken on a per household basis, at a density of 1:3 to 1:10 houses. Between 2016 and 2017, releases were undertaken on an area basis, where the target release area was divided into a series of 100 m × 100 m grids, with a single release point located inside each grid. Adult mosquitoes were transported to the field in release cups in vehicles and released on a single day each week, normally at the property line or front yard by removing the container lids and gently shaking. Releases were generally undertaken between 0800–1600 hours each day.

Between 2011 and 2013 the duration of releases was fixed to between 9 and 16 weeks, except for an initial trial of egg releases in Stratford (SF3) where the methodology was being optimized over a longer period (23 weeks). Shorter duration release periods (7- or 8-weeks) were trialed in Charters Towers and Douglas Shire in 2016, and these were extended in Tully and Innisfail (Cassowary Coast) to 12-week releases and 14- to 16-week releases, respectively, in 2017. The 2016–2017 Cairns releases were staged, with releases continuing in a release area until the frequency of *Wolbachia* in samples of field-caught mosquitoes was above 50% for two consecutive weeks. The duration of releases in these areas varied from 10–12 weeks, except for Cairns North Ext (CNX) where only two weeks of releases were undertaken. Releases in this area were discontinued after this time because the *Wolbachia* infection frequency in mosquitoes already exceeded 90% following the spread of *Wolbachia* from nearby release areas. Details of the releases in each area are summarized in Table 1.

### Direct egg releases

**Figure 4.**
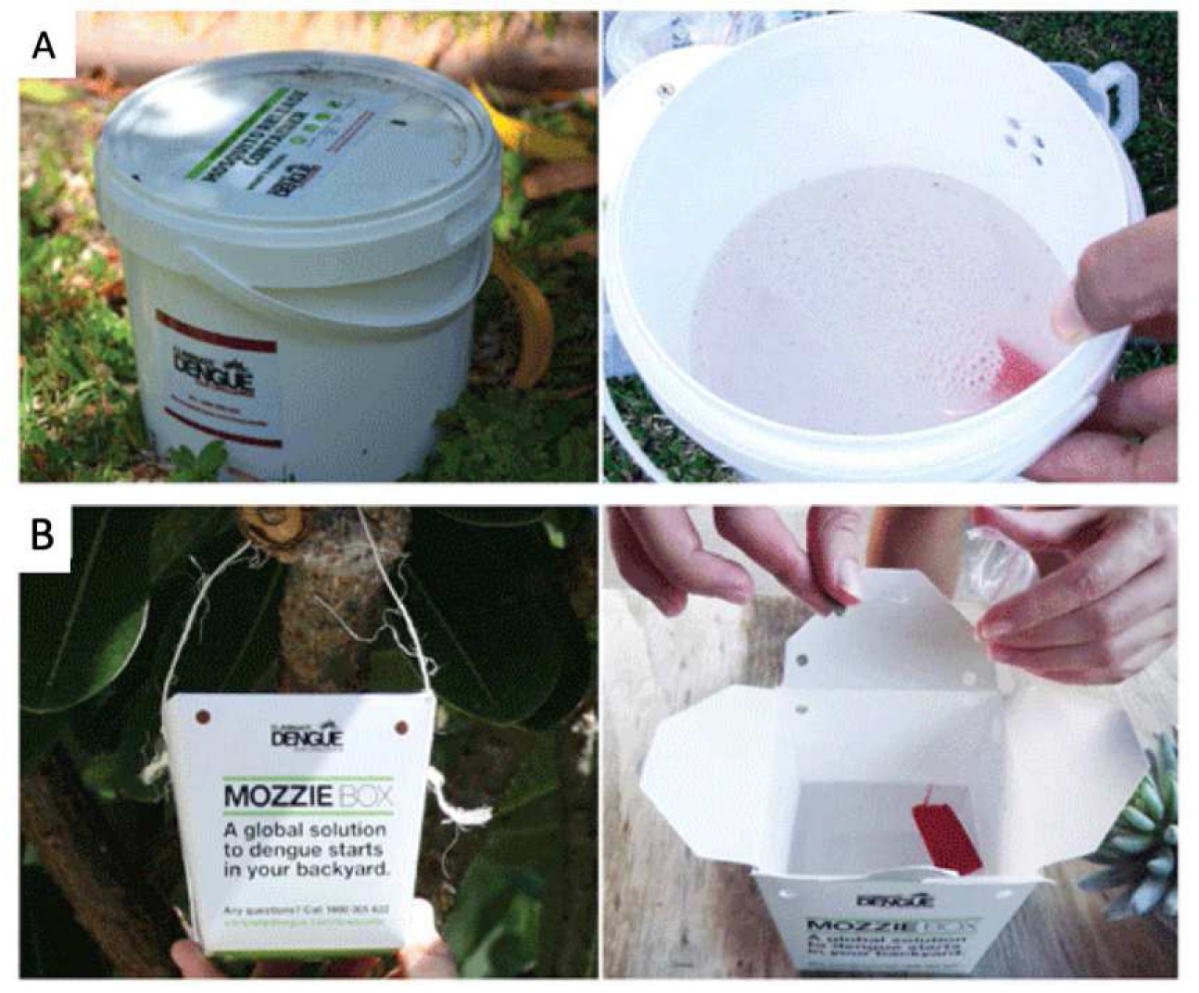
Mosquito-release containers. Photos illustrating different mosquito release containers used in the deployment. Bucket mosquito release container (MRC) ( **A**) Single use Mozzie Box MRC ( **B**) ( O’Neill *et al.*, 2018).

Small-scale field trials to develop and optimize the egg release methods were undertaken in parts of Babinda and Machans Beach in 2013, and in Bungalow (BU1–3), Cairns North (CN1) and Stratford (SF1–3) in 2014. For the 2013 trials, 3 L white polypropylene buckets with lids (Piber Plastics, Australia) were used ( Figure 4A). Each bucket had four 6 mm holes drilled 20 mm apart in a square pattern in the side. Tangle-Trap Sticky Coating (Tanglefoot, USA, Product number 300000588) was applied around the perimeter of the emergence holes (1–2 cm away from holes) to prevent the entry of ants into the buckets. The buckets interiors were roughened with sandpaper to provide perching for mosquitoes post-emergence. Containers were filled with 2 L of tap water and 1 lucerne pellet (0.5 g) (LLP, Carole Park, Australia) and 1 TetraMin Tropical Tablet (broken into four pieces) were added, along with an egg strip containing approximately 150 eggs (target emergence rate of 100 adults per container). Containers were placed in a shaded position near the front boundary of selected houses. Containers were serviced every 2 weeks, at which time live/dead larval/pupal counts were made and the general condition of the container (tipped over, dry/empty, fouled water) was recorded for quality assurance. During the cooler months, immature development in some containers was slowed and necessitated leaving containers in place for 3 weeks prior to servicing. Once immature development was complete, the remaining contents of the containers was discarded and the inside of the container was cleaned with a sponge to remove any residual eggs and larvae/pupae. The container was then refilled with clean tap water, and new food and eggs were added as described above.

The 2014 egg release trials were similar to the above, except these involved 2.3 L plastic buckets containing 1 L of tap water. An alternative feeding mixture involving ground red kidney beans (1.25 g per container) was used in these trials. An assessment of egg hatch rate (hatch rate quality assurance) was undertaken in the laboratory prior to each release round and this information was used to calculate the required numbers of eggs to be added to each container. The target emergence rate for these trials was 75 adults per container.

For the 2015 egg releases, the conditions were the same as the 2014 releases except Aqua One Vege Wafers were used as a food source (4 wafers per containers in spring/summer/autumn months, and 5 wafers per containers in winter months), and each container received 100 viable eggs).

### Community and school egg releases

Community egg releases undertaken by school children (Bentley Park 2016, Charters Towers 2016, Mission Beach 2016) and local council staff and Rotary group members (Douglas Shire 2016) involved single use “Mozzie Box” containers which consisted of a 775 ml food container (Detpak, Australia) without handle, and with measurements of 104 × 92 mm (top), 79 × 61 mm (base), 104 mm (height) ( Figure 4B, O’Neill *et al.*, 2018). Four holes were punched into each container and 400ml of water was added to each container. Each Mozzie Box container received food and eggs as described above for 2015 egg releases.

### Field monitoring

Mosquito collections were undertaken during and after releases using BG Sentinel (BGS) traps (Biogents AG, Regensburg, Germany, Product number NR10030). Mosquitoes were collected and returned to the laboratory for sorting, morphological identification and counting. *Aedes aegypti* samples were stored in 70% ethanol prior to screening for *Wolbachia* infection status. BGS trap samples were collected each week during releases, and every one to two after releases. Routine BGS trap collections were maintained for between 2–18 months post release, except for Charters Towers where BGS traps were withdrawn three weeks after completion of releases. After this time BGS traps were reinstalled periodically every 6–12 months and samples were collected after 1–2 weeks.

The number and density of BGS traps in each area varied across the different release periods. Monitoring of releases up until 2014 involved relatively high numbers of BGS traps per area, ranging from 11–80 traps per area and equivalent to 27–160 traps per km ^2^. From 2015, as release areas increased in size, BGS monitoring was undertaken on a per area basis, with densities of 8 BGS traps per km ^2^ during 2015 in Cairns, and this was further reduced to 4 BGS traps per km ^2^ from 2016 onwards in Cairns, Charters Towers and the Cassowary Coast. *Ae. aegypti* counts and *Wolbachia* screening results, aggregated by release area and collection period are available as *Underlying data* ( Ryan, 2019).

### Diagnostics

Adult *Ae. aegypti* were screened for *Wolbachia* using Taqman qPCR on a Roche LightCycler 480 using an internally controlled qualitative assay for the presence or absence of *Wolbachia* as previously described ( Dar *et al.*, 2008; O’Neill *et al.*, 2018; Yeap *et al.*, 2014). The qPCR cycling program consisted of a denaturation at 95°C for 5 min followed by 45 cycles of PCR (denaturation at 95 °C for 10 sec, annealing at 60 °C for 15 sec, and extension at 72 °C for 1 sec with single acquisition) followed by a cooling down step at 40°C for 10 sec. From September 2018 *Wolbachia* diagnostics were performed by LAMP. LAMP reactions were performed in a Bio-Rad C1000 96-well PCR thermocycler with a 30min incubation at 65°C as previously described ( O’Neill *et al.*, 2018). Individual reactions consisted of 2X WarmStart® Colorimetric LAMP Master Mix (New England BioLabs, Cat# M1800S), primers and 1 μL of target DNA in a total reaction volume of 17 μL. Reactions for individual samples were performed in 96-well PCR plates (LabAdvantage 96-well PCR plates, full skirt, clear). Plates were incubated in a thermocycler (BioRad C1000) at 65°C for 30 minutes then held at 12°C until scoring. Within one hour of incubation, colour changes of individual samples were recorded. Primers were as follows FIP 5’ TGTATGCGCCTGCATCAGCTTCGGTTCTTATGGTGCTAA, BIP 5’ GCAGAAGCTGGAGTAGCGTTGTGTCATGCCACTTAGATGG, F3 5’ TGATGTAACTCCAGAAGTCA, B3 5’ CTTATTGGACCAACAGGATCG, LpF 5’ AGCCTGTCCGGTTGAATT, LpB 5’ CAGTCTTGTTATCCCAGTGAGT.

### Dengue case notification data

Dengue is a notifiable disease in Australia, which mandates clinicians and laboratories to report confirmed and suspected cases to local health authorities. De-identified data was provided by the Queensland Health Communicable Diseases Branch on all laboratory-confirmed and clinically diagnosed (probable) dengue cases notified from Townsville and Cairns and Hinterland Hospital and Health Services (THHS and CHHHS) to the Notifiable Conditions System (NoCS) between 1 January 2000 and 31 March 2019. The NoCS case records include a variable indicating whether the case was classified as imported, on the basis of a history of overseas travel during the 3–12 days prior to illness onset, or locally-acquired. This information is routinely captured in case notifications based on interview by local public health units.

The Townsville Public Health Unit (PHU) provided line-listed data on addresses of all dengue cases notified from THHS and CHHHS, from the operational databases of the Cairns Tropical Public Health Service and the Townsville PHU. This data included one or more addresses per case, with an indication of the primary residential address, and of any address that had been classified as the probable location of dengue acquisition or a possible site of exposure, during the course of public health follow up. We linked the PHU dataset to the NoCS dataset by unique case notification identifier. All NoCS records were retained, with or without a linked PHU record. PHU records without a linked NoCS record were excluded, as NoCS is considered the master source of case notifications data, and holds the travel history variable to distinguish locally-acquired from imported cases.

Approval to access anonymized spatially identifiable dengue case notification data, collected as part of routine disease surveillance, was obtained from the Townsville Hospital and Health Service human research ethics committee (HREC/16/QTHS/108) and research governance office (SSA/16/QTHS/238), and from the Office of the Director-General, Queensland Department of Health, under the Public Health Act 2005.

### Classification of *Wolbachia* exposure status

A case’s location, for the purpose of classifying *Wolbachia* exposure status, was determined using geolocatable address information from the PHU operational database. The address indicated in the PHU dataset as the probable location of dengue acquisition was used where available (53%); if unavailable then the primary residential address was used (22%). For 20% of cases a geolocatable address was available in the operational database but was not designated as either ‘acquired’ or ‘residential’, and for the remaining 4% no geolocatable address was available in the PHU database, and the suburb of residence from the NOCS dataset was used to define the case’s designated location. Case geolocations were overlaid with *Wolbachia* release area boundaries in ArcMap (version 10.5, ESRI, Redlands, USA) (QGIS is an open-access alternative) to classify the *Wolbachia* exposure status of each case at the time of case onset. An area was considered exposed (post-intervention) from the date of last release. Five ‘non-release areas’ in central Cairns, where BGS monitoring indicated that *Wolbachia* had established by dispersal from neighboring release areas, were considered exposed from the inferred date that the *Ae. aegypti* infection curve crossed 80%. The local government area (LGA) of each case was determined from its location, as classified above, and any cases located outside of Cairns, the Cassowary Coast, Charters Towers or Douglas LGAs were excluded from the analysis.

### Population data

The populations of each release area and *Wolbachia*-exposed non-release area ( Figure 1– Figure 3) were estimated by aggregating from mesh blocks (ABS, 2016) to the boundaries of each intervention area ( Table 1), in ArcMap. In all but three release areas, the boundaries of the release area and monitored area were aligned, and defined the *Wolbachia*-exposed population for the purpose of epidemiological analyses. The exceptions were Gordonvale, Yorkeys Knob, and Babinda, where monitoring extended beyond the boundaries of the release area and demonstrated *Wolbachia* establishment throughout this extended area. The population denominator for these three areas was therefore calculated for the larger monitored area ( Table 1 footnote). A further exception was Stratford, where concurrent releases were conducted in three small non-contiguous areas, which represented ~75% of the area and 90% of the population of the suburb of Stratford. For the purpose of epidemiological analysis, the whole suburb population was considered *Wolbachia*-exposed from the completion of the last releases in the three release zones. For the interrupted time series analysis, monthly aggregate treated and untreated areas (and their resident populations) were calculated, dynamic over time, with the treated area in any given month defined as the total area where *Wolbachia* deployments had been completed to date (or where, for the five central Cairns non-release areas where *Wolbachia* established, the inferred local *Wolbachia* frequency had reached 80%, as above).

### Statistical methods

Locally-acquired and imported dengue case notifications were cross-tabulated by month of illness onset and LGA. Scaled ‘time-since-release’ (TSR) was calculated for each case located within a *Wolbachia* exposed area, as (date of case onset – date of last release in the local release area) rounded to the nearest month. TSR is equal to zero for cases with onset in the same month as releases were completed, and is positive for cases with onset post-intervention. Cases located in *Wolbachia* non-exposed areas were excluded from the TSR analysis. The staggered deployment of *Wolbachia* across release areas from January 2011 to May 2017 means the distribution of pre-intervention and post-intervention time within the dengue case time series (Jan 2000 – Mar 2019) is variable between release areas. For each release area, the pre-intervention period was calculated as months from January 2000 to end of releases, and post-intervention period as months from end of releases to March 2019. Locally-acquired and imported dengue case notifications were tabulated by month of TSR, and summary statistics for the distribution of pre- and post-intervention time were calculated.

To better visualize the temporal distribution of locally-acquired and imported dengue case notifications with respect to *Wolbachia* deployments, release areas were grouped by calendar quarter of last release, and cases were plotted by release area group against date of illness onset, stratified by locally-acquired vs imported cases.

Negative binomial regression was used to model monthly counts of locally-acquired dengue cases (January 2000 – March 2019) in aggregate *Wolbachia*-treated and not-yet-treated areas. Cases located in *Wolbachia* non-exposed areas were excluded from the analysis. The regression model was fitted in Stata (SE version 14.2, StataCorp, TX) using generalized estimating equations, with epidemic year (September – August) as the cluster variable to account for temporal autocorrelation in the monthly case counts, adjusting for monthly imported dengue cases (any vs none) and season (dry: June – November vs wet: December – May), with a population size offset. A binary intervention variable was included in the regression model to distinguish the pre- or post-intervention status of each area in any given month, the coefficient of which provided the estimate of intervention effect (incidence rate ratio).

### Ethical considerations and consent

Regulatory approval for the release of *Aedes aegypti* containing *Wolbachia* was provided by the Australian Pesticides and Veterinary Medicines Authority (Permit numbers 12311, 13183, 13718, 13810, 14266, 14762, 14530, 82947, 14762).

Ethics approval for human blood feeding mosquito colonies in Melbourne was issued from Monash University (CF11/0766 a 2011000387). All volunteers (no children involved) provided written consent.

In Cairns, Human Ethics approval for bloodfeeding was provided by Human Research Ethics Committee, James Cook University (H4907). All adult subjects provided informed oral consent (no children were involved). Names of subjects providing oral consent were recorded in writing.

Verbal and/or written consent from participants was obtained by phone, online or face-to-face to set BG traps, set MRCs, or participate in Community Mosquito Releases.

Surveys were undertaken under Monash ethics: CF13/2407 – 2013001272 Community knowledge of dengue and *Wolbachia* based dengue control and CF13/2805 – 2013001515 Community knowledge of dengue and *Wolbachia* based dengue control – Townsville.

Approval to access anonymized spatially identifiable dengue case notification data, collected as part of routine disease surveillance, was obtained from the Townsville Hospital and Health Service human research ethics committee (HREC/16/QTHS/108) and research governance office (SSA/16/QTHS/238), and from the Office of the Director-General, Queensland Department of Health, under the Public Health Act 2005.

## Results and discussion

**Figure 5.**
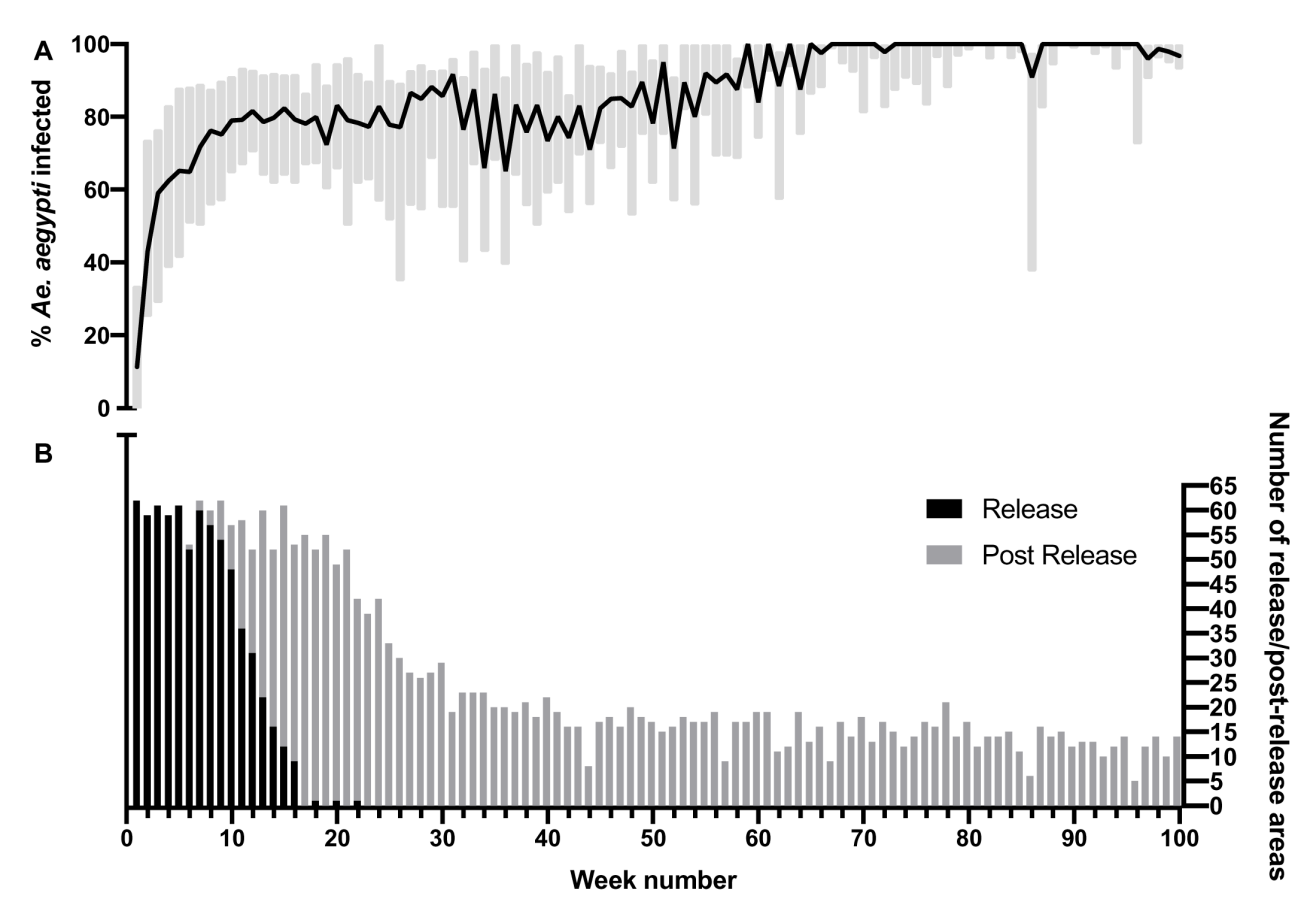
*Wolbachia* infection rates in *Aedes aegypti* mosquitoes collected from individual release areas during release and post-release monitoring periods. Line represents median percentage infection rate across 62 individual release areas, box represents interquartile range, Week number 1 corresponds to commencement of *Wolbachia* mosquito releases in each area. ( **A**), Number of release and post-release areas monitored each week ( **B**).

Overall, there was a predictable and consistent trajectory of *Wolbachia* establishment in *Ae. aegypti* populations as a result of relatively short term (median release duration 12 weeks, range 5–23 weeks), low density releases of *Wolbachia* infected mosquitoes (generally 20% of houses), either as adults or eggs, across a variety of communities in Cairns and surrounding areas in northern Australia ( Figure 5). *Wolbachia* frequency data from mosquitoes collected during release and post-release monitoring periods were compiled by week (week 1 = first release) for each release area (non-release areas as per Table 1 were excluded from the analyses, along with the following release areas KB, CNX, EHX, MRAX, BUX3, AER and FW as the frequency of *Wolbachia* in mosquitoes in week 1 was already high as a result of likely spread of *Wolbachia* from nearby release areas). By week 12 of releases, the median *Wolbachia* frequency in mosquitoes was 82.4% and this increased to 92.3% by week 22. After completion of releases (>23 weeks), median weekly *Wolbachia* frequencies ranged between 66.0–95.0% through until week 52, and were above 80% thereafter.

**Figure 6.**
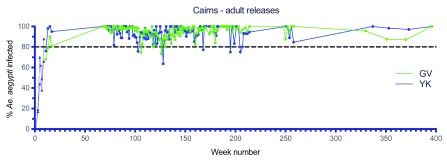
*Wolbachia* infection rates in *Aedes aegypti* mosquitoes collected from Gordonvale (GV) and Yorkeys Knob (YK) during release (triangles) and post-release (circles) monitoring periods. Collections to week 17 from ovitraps, collections from BG Traps thereafter ( *Wolbachia* infected adult mosquito releases undertaken weekly for 10 weeks between Jan–Feb 2011, Week number 1 corresponds to commencement of *Wolbachia* mosquito releases in each location).

**Figure 7.**
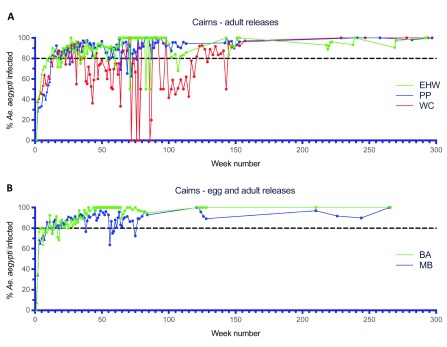
*Wolbachia* infection rates in *Aedes aegypti* mosquitoes collected from Edge Hill/Whitfield (EHW), Parramatta Park (PP) and Westcourt (WC) ( **A**) and Babinda (BA) and Machans Beach (MB) ( **B**) during release (triangles) and post-release (circles) monitoring periods ( *Wolbachia* infected adult releases were undertaken weekly for 15–16 weeks between Jan–Apr 2013 in EHW, PP and WC; *Wolbachia* infected adult and egg stage releases undertaken weekly for 9–11 weeks between Jul–Sep 2013 in BA and MB, Week number 1 corresponds to commencement of *Wolbachia* mosquito releases in each location).

Longitudinal monitoring of the *Wolbachia* infection frequency in mosquitoes collected from the initial Yorkeys Knob and Gordonvale release sites ( Hoffmann *et al.*, 2011; Hoffmann *et al.*, 2014) indicated that *Wolbachia* has been maintained in the mosquito populations at high levels with mean *Wolbachia* frequencies of 94.7% and 95.4%, respectively, for over 8 years ( Figure 6). Similar results from releases undertaken in 2013 in inner Cairns suburbs ( Schmidt *et al.*, 2017) ( Figure 7A) indicated that once *Wolbachia* had been established in local mosquito populations, it persisted at high levels, even in contiguous urban landscapes that were surrounded by areas with *Wolbachia* uninfected *Ae. aegypti* populations. Despite initial fluctuations in *Wolbachia* frequency in the suburb of Westcourt ( Schmidt *et al.*, 2017), a small release site of approximately 0.1 km ^2^ in size, *Wolbachia* eventually established in the mosquito population without any additional releases ( Figure 7A). *Aedes aegypti* counts and *Wolbachia* screening results, aggregated by release area and collection period are available as *Underlying data* ( Ryan, 2019).

**Figure 8.**
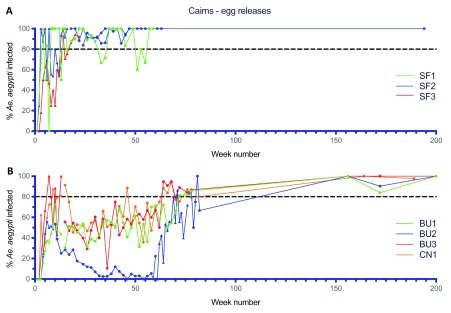
*Wolbachia* infection rates in *Aedes aegypti* mosquitoes collected from Stratford 1-3 (SF1-3) ( **A**) and Bungalow 1-3 (BU1-3) and Cairns North (CN) ( **B**) and during release (triangles) and post-release (circles) monitoring periods ( *Wolbachia* infected egg releases were undertaken weekly for 4, 16 and 19 weeks in SF1-3, respectively, from Jun–Nov 2014, and for 12 and 13 weeks in BU1 and BU3, respectively, from Aug–Oct 2014; BU2 had two rounds of egg releases – 8 weekly releases from Aug–Sep 2014, followed by 12 weekly releases from Oct–Dec 2015, Week number 1 corresponds to commencement of *Wolbachia* mosquito releases in each location).

**Figure 9.**
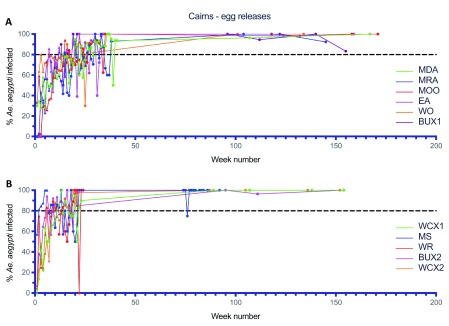
*Wolbachia* infection rates in *Aedes aegypti* mosquitoes collected from Manunda (MDA), Manoora (MRA), Mooroobool (MOO), Earlville (EA), Woree (WO) and Bungalow Ext 1 (BUX1) ( **A**), Westcourt Ext 1 (WCX1), Mount Sheridan (MS), White Rock (WR), Bungalow Ext 2 (BUX2) and Westcourt Ext 2 (WCX2) ( **B**) during release (triangles) and post-release (circles) monitoring periods ( *Wolbachia* infected egg releases were undertaken every 2 weeks for 9–15 weeks in MDA, MRA, MOO, EA, WO and BUX1 between May–Dec 2015, and for 6–13 weeks in WCX1, MS, WR, BUX2 and WCX2 between Sep–Dec 2015), Week number 1 corresponds to commencement of *Wolbachia* mosquito releases in each location).

Early deployments of *Wolbachia* involving egg releases, either in combination with adult mosquito releases ( Figure 7B) or on their own ( Figure 8), allowed testing and development of the egg release methods, and also calibration of release rates both in terms of the density and the duration of releases. Egg releases into small isolated sites (SF1–3), involving weekly releases at 20% of houses for between 5-23 weeks all resulted in successful establishment of *Wolbachia* ( Figure 8A). Egg releases into four areas with higher populations of *Ae. aegypti* (BU1–3 and CN1) at similar or lower release densities (5–10% of houses), resulted in the slower establishment of *Wolbachia* in three of these areas ( Figure 8B, BU1 and BU3, > 1 year to reach 80% frequency). In contrast, in BU2 where the *Wolbachia* frequency only reached 50% after 9 weeks of releases, the frequency of *Wolbachia* declined to less than 10% after week 30 ( Figure 8B). The long-term decline in *Wolbachia* frequencies in mosquitoes in BU2 suggests that releases in this area did not result in *Wolbachia* exceeding the threshold frequency of infection, above which frequencies systematically increase ( Schmidt *et al.*, 2017). Given the decline in *Wolbachia* frequencies, egg releases re-commenced at week 62 for 16 weeks and resulted in *Wolbachia* establishment. Larger, operational scale egg releases were undertaken across the remaining inner Cairns areas in 2015 ( Figure 9) (11.5 km ^2^; 13,823 households) and involved a fixed density of releases at 20% of households, but with variable durations of releases of between 6-15 weeks (mean 11.5 weeks). *Wolbachia* frequencies in mosquitoes at the end of releases in each area ranged from 67.4 to 100%. Periodic monitoring of mosquitoes from these areas between weeks 75–171 indicated that the *Wolbachia* frequency in mosquitoes was high across all sites (mean 98.7%, range 75–100%).

**Figure 10.**
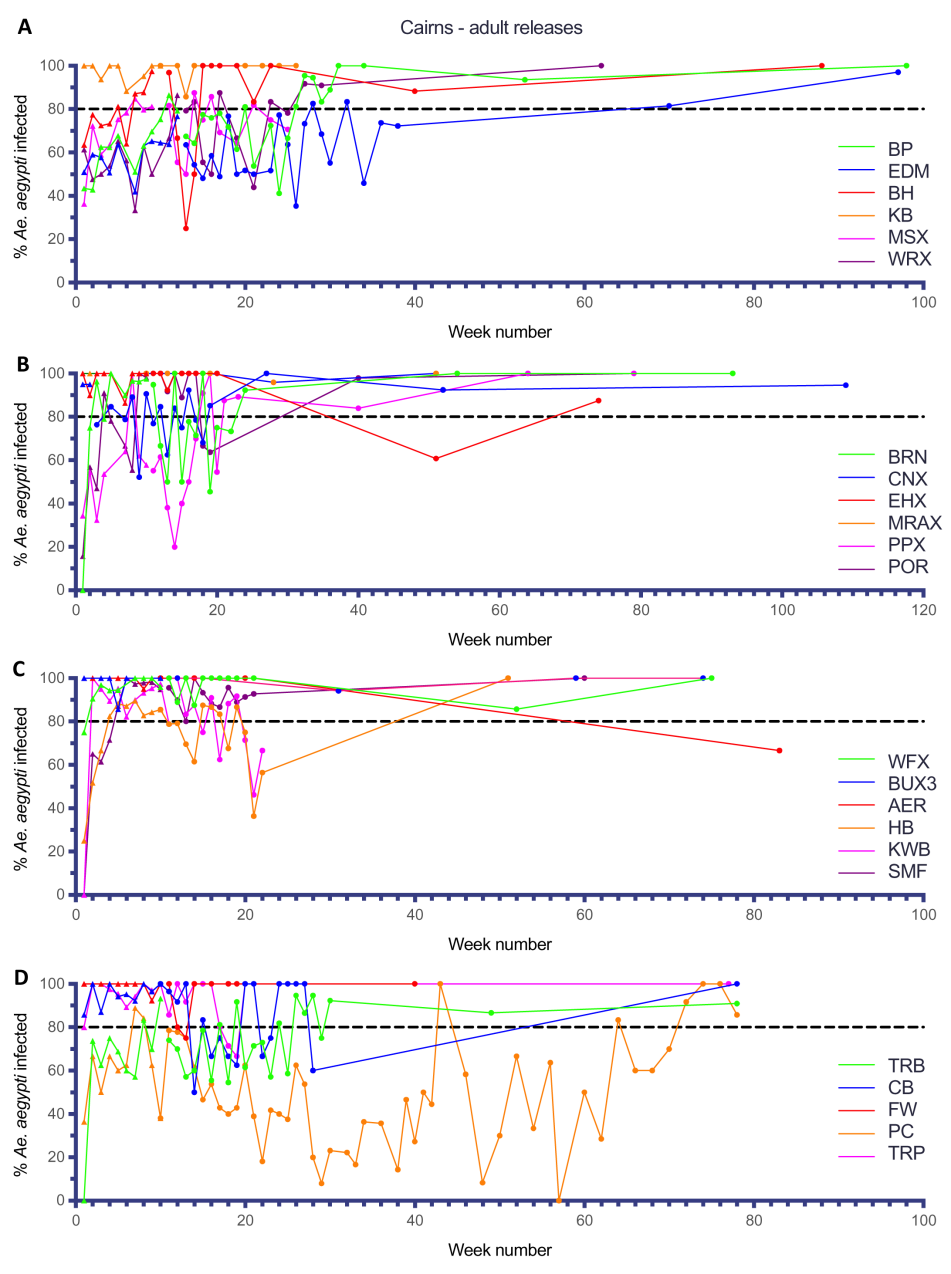
*Wolbachia* infection rates in *Aedes aegypti* mosquitoes collected from Bentley Park (BP), Edmonton (EDM), Bayview Heights (BH), Kanimbla (KB), Mount Sheridan Ext (MSX) and White Rock Ext (WRX) ( **A**), Brinsmead (BRN), Cairns North Ext (CNX), Edge Hill Ext (EHX), Manoora Ext (MRAX), Parramatta Park Ext (PPX) and Portsmith (POR) ( **B**), Whitfield Ext (WFX), Bungalow Ext 3 (BUX3), Aeroglen (AER), Holloways Beach (HB), Kewerra Beach (KWB) and Smithfield (SMF) ( **C**), Trinity Beach (TRB), Clifton Beach (CB), Freshwater (FW), Palm Cove (PC) and Trinity Park (TRP) ( **D**) during release (triangles) and post-release (circles) monitoring periods ( *Wolbachia* infected adult releases were undertaken every week for 9–11 weeks in BP, EDM, BH, KB, MSX and WRX between Nov 2016 and May 2017, for 3–9 weeks in BRN, CNX, EHX, MRAX, PPX and POR between Mar–May 2017, for 5–10 weeks in WFX, BUX3, AER, HB, KWB and SMF between Mar–Jul 2017, for 9–10 weeks in TRB, CB, FW, PC and TRP between May–Aug 2017), Week number 1 corresponds to commencement of *Wolbachia* mosquito releases in each location).

Large scale adult mosquito releases were undertaken across the remaining Cairns suburbs (46.4 km ^2^, 35,899 households), the Cassowary Coast (12.2 km ^2^, 7,940 households), Charters Towers (6.9 km ^2^, 3,359 households) and Douglas Shire (7.1 km ^2^, 2,585 households) between 2015–2017 ( Figure 10– Figure 14). Similar patterns were observed in terms of *Wolbachia* establishment in Cairns, Charters Towers and Douglas Shire releases, although frequencies were more variable in Palm Cove (PC) where the *Wolbachia* frequency in mosquitoes didn’t reach 80% until after week 72 ( Figure 10D). Releases in the Cassowary Coast – Innisfail area were undertaken between Mar-Jun 2017 and coincided with a period of generally low *Ae. aegypti* adult mosquito numbers. *Wolbachia* frequencies in mosquitoes after 14–16 weeks of releases were relatively high (mean 67.8%, range 50.0-100.0%) across the releases areas, although this was followed by a drop in *Wolbachia* frequencies and high variability between weeks 20–40 (mean 58.1%, range 30.0–80.2%) ( Figure 13). In three of these areas (subarea in Innisfail, Mourilyan and South Johnston) the Verily Debug project undertook releases of *w*AlbB infected male *Ae. aegypti* mosquitoes which were expected to induce sterility when mated to *w*Mel infected females and well as uninfected females ( Axford *et al.*, 2016). Release dates were not stated, however *w*AlbB infected male *Ae. aegypti* mosquitoes were detected in BGS trap collections between weeks 39–66 in Mourilyan and weeks 41–66 in Innisfail and South Johnstone ( Figure 13B). The release of *w*AlbB male *Ae. aegypti* mosquitoes coincided with a period when there was a drop in *w*Mel *Wolbachia* infection frequency in mosquitoes in Mourilyan (mean 61.5% between weeks 39–66), although the *w*Mel *Wolbachia* frequency increased and was maintained generally above 80% from week 55 onwards. There was no appreciable effect of *w*AlbB male *Ae. aegypti* releases on the *w*Mel frequency in either Innisfail or South Johnstone ( Figure 13B). *Wolbachia* ( *w*Mel) infection frequencies in mosquitoes across the three *w*AlbB release areas were high from weeks 70 onwards (mean 94.0%, range 85.7–97.0%), indicating that *w*Mel *Wolbachia Ae. aegypti* persisted in these areas, despite the release of relatively large numbers (3 million, Verily Debug project) of incompatible *w*AlbB male *Ae. aegypti* mosquitoes.

**Figure 11.**
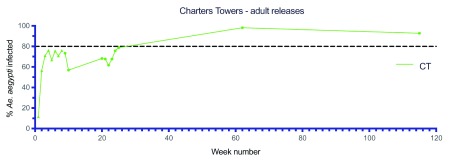
*Wolbachia* infection rates in *Aedes aegypti* mosquitoes collected from Charters Towers (CT), during release (triangles) and post-release (circles) monitoring periods ( *Wolbachia* infected adult releases were undertaken every week for 8 weeks between Oct–Nov 2016, Week number 1 corresponds to commencement of *Wolbachia* mosquito releases).

**Figure 12.**
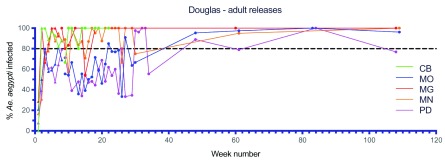
*Wolbachia* infection rates in *Aedes aegypti* mosquitoes collected from Cooya Beach (CB), Mossman (MO), Mossman Gorge (MG), Mossman North (MN) and Port Douglas (PD), during release (triangles) and post-release (circles) monitoring periods ( *Wolbachia* infected adult releases were undertaken every week for 7–8 weeks between Oct–Dec 2016, Week number 1 corresponds to commencement of *Wolbachia* mosquito releases).

**Figure 13.**
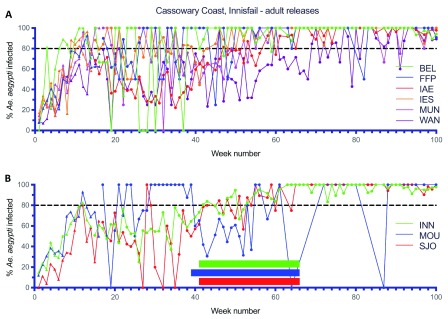
*Wolbachia* infection rates in *Aedes aegypti* mosquitoes collected from Belvedere (BEL), Flying Fish Point (FFP), Innisfail East (IAE), Innisfail Estate (IES), Mundoo (MUN) and Wangan (WAN) ( **A**) and Innisfail (INN), Mourilyan (MOU) and South Johnstone (SJO) ( **B**) during release (triangles) and post-release (circles) monitoring periods ( *Wolbachia* infected adult releases were undertaken in BEL, FFP, IAE, IES, MUN, WAN, INN, MOU and SJO every week for 14–16 weeks between Mar–Jun 2017, Week number 1 corresponds to commencement of *Wolbachia* mosquito releases). In INN, MOU and SJO, *w*AlbB infected male-only *Ae. aegypti* mosquito releases were undertaken between weeks 41–66 in INN and SJO, and between weeks 39–66 in MOU. Shaded horizontal bars correspond to *w*AlbB male release period. Estimation of weekly *w*Mel *Wolbachia* mosquito infection rates excluded *w*AlbB males from the calculation.

**Figure 14.**
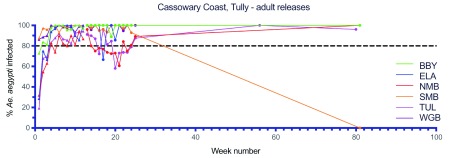
*Wolbachia* infection rates in *Aedes aegypti* mosquitoes collected from Bingal Bay (BBY), El Arish (ELA), North Mission Beach (NMB), South Mission Beach (SMB), Tully (TUL) and Wongaling Beach (WGB) during release (triangles) and post-release (circles) monitoring periods ( *Wolbachia* infected adult releases were undertaken every week for 12 weeks between May–Aug 2017, Week number 1 corresponds to commencement of *Wolbachia* mosquito releases).

Overall, short-term releases of between 5–23 weeks involving either egg or adult stages, resulted in the establishment of *Wolbachia* in mosquito populations across all release areas. There were no clear differences between egg or adult releases in terms of *Wolbachia* establishment. The overall duration of egg and adult release periods were similar (average 12 weeks duration for both egg and adult releases), although egg releases were generally undertaken every 2 weeks compared with weekly adult mosquito releases. Although *Ae. aegypti* populations varied seasonally across the study areas, there were no clear seasonal effects on *Wolbachia* establishment, indicating that under the north Queensland conditions releases can be undertaken year round. Operationally, egg releases provided advantages over adult mosquito releases in that there was no need to rear immatures stages to adults in a local insectary. For egg releases, eggs were produced centrally in an insectary, and then transferred to the field and placed into mosquito release containers, either by staff or via community members themselves. These simple, low-cost egg release methods may represent a more scalable approach for future large-scale implementations, particularly in low resource settings where infrastructure for mass rearing of adult mosquitoes is limited.

**Figure 15.**
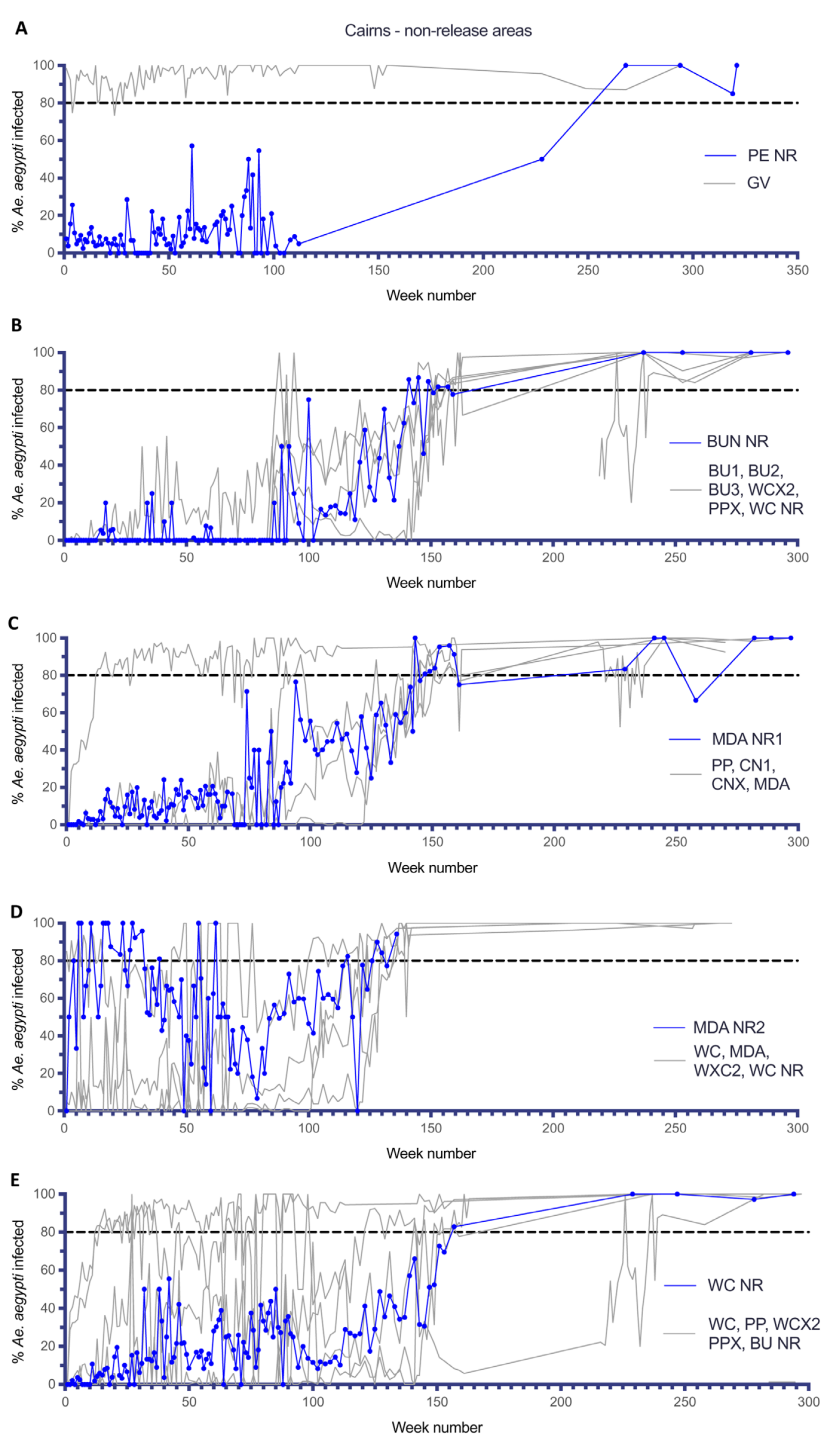
*Wolbachia* infection rates in *Aedes aegypti* mosquitoes collected from Pyramid Estate non-release area (PE NR) ( **A**), Bungalow non-release area (BUN NR) ( **B**), Manunda non-release area 1 (MDA NR1) ( **C**), Manunda non-release area 2 (MDA NR2) ( **D**) and Westcourt non-release area (WC NR) ( **E**) non-releases areas. Week number 1 corresponds to commencement of *Wolbachia* mosquito monitoring in each non-release area (PE NR week 1 = 21/12/2012, BUN NR week 1 = 11/01/2013, MDA NR1 week 1 = 11/1/2013, MDA NR2 week 1 = 07/06/2013, WC NR week 1 = 11/01/2013). Grey lines show corresponding weekly *Wolbachia* infection rates in *Aedes aegypti* mosquitoes collected from adjacent release or non-release areas (areas described in Figure 2 and Table 1).

Previous analyses of the spatial spread of *Wolbachia* from the 2013 inner Cairns release sites (EHW, PP and WC, Figure 2A) indicated that spread of *Wolbachia* from the release sites were spatially heterogeneous, *Wolbachia* moved relatively slowly at 100–200m per year ( Schmidt *et al.*, 2017), and this was possibly due to barriers to *Ae. aegypti* dispersal, higher incidence of long-range *Ae. aegypti* dispersal, and intergenerational loss of *Wolbachia* ( Schmidt *et al.*, 2018). The current analyses of *Wolbachia* infection frequencies in mosquitoes from five non-release areas ( Figure 2A, 2B, Figure 15) indicated that *Wolbachia* became established in mosquito populations throughout each area. In the case of the Pyramid Estate non release area (PE NR) which was located west of a main highway which separated it from the initial Gordonvale release site ( Figure 2B), *Wolbachia* infection frequencies remained low (<20%) for over 100 weeks, despite the high (>80%) *Wolbachia* frequency in mosquitoes in Gordonvale during the same period ( Figure 15A). Periodic monitoring of mosquitoes in Pyramid Estate at week 228 indicated that the *Wolbachia* frequency in mosquitoes had reached 50%, and had further increased to above 80% from week 268 onwards. Although monitoring was only undertaken periodically in Pyramid Estate from week 100, the increase in the *Wolbachia* frequency in this area, despite a relatively low frequency during the first 100 weeks, suggests that the natural introduction of *Wolbachia* mosquitoes, as either eggs or adults from nearby Gordonvale, was sufficient to result in eventual establishment of *Wolbachia*. Similar results were found in the four other non-release sites, although in each of these sites the *Wolbachia* infection frequency was generally correlated with the *Wolbachia* infection frequency in mosquitoes in nearby release sites ( Figure 15B-E). In these four inner Cairns non-release sites there were only limited boundaries to mosquito movement, and once *Wolbachia* became established in nearby release areas the infection spread into mosquitoes in nearby non-release areas. Overall, these five non-release areas constituted 3.23 km ^2^ and some 3,261 households, and indicated that releases of *Wolbachia* mosquitoes do not need to be undertaken in all areas where *Ae. aegypti* occur ( Turelli & Barton, 2017). This opens the way for more efficient deployment strategies in the future where areas are left intentionally with no mosquito releases and instead rely on natural spreading of *Wolbachia.* This natural spreading may take some time depending on the size of deliberate deployment “holes” and local variation in mosquito density and mosquito habitat ( Hancock *et al.*, 2019) but has the potential to significantly reduce deployment costs. Moreover, these non-release areas can be considered in the vertical dimension and not just the horizontal dimension given the nature of dispersal of *Ae. aegypti* within buildings ( Liew & Curtis, 2004). In this scenario deployments in upper floors of collections of high-rise apartment buildings may not be needed, instead relying on *Wolbachia* to naturally invade these areas, simplifying deployment logistics.

In the Cairns releases between 2011–2014, communication and community engagement activities followed earlier approaches described in Hoffmann *et al.* (2011), and relied heavily on face-to-face consultation with key stakeholders and community groups, including one-on-one meetings, attendance at community events, door-knocking and mail-outs to householders. This proved effective in building awareness of the project and generated support for and participation in releases. From 2015 as release activities scaled up to cover larger areas of Cairns, the PAM was used for community engagement, and this proved effective in building awareness of the project and broad support for activities ( Table 2). Community members volunteered to participate in activities and this lead to a pre-registered participant database through which field staff could distribute mosquito release containers and mosquito monitoring traps. In addition to hosting mosquito release containers and mosquito monitoring traps, local ownership of the WMP’s *Wolbachia* method was achieved through a school program conducted at Bentley Park College in 2017. Known as the Wolbachia Warriors Program ( O’Neill *et al.*, 2018), the voluntary, applied-science program was undertaken by 636 students aged from five to 12 years of age. The free program involved the engagement of teachers, parents and students to enable participants to grow and release *Wolbachia* carrying mosquitoes in their yards at home, three times, over six weeks. Each participant was provided with an instructive project booklet and three Mozzie Boxes (mosquito egg release kits). By participating in an applied science program, students learnt basic natural history that complemented in-class learning, while directly contributing to public health outcomes.

Similar to the Cairns releases above, the PAM model was also implemented in Charters Towers, Douglas Shire and the Cassowary Coast. This was implemented prior to releases and included the same components as in Cairns ( Table 2), and generated significant awareness and support, and direct participation of communities in releases:

- Charters Towers – the Wolbachia Warriors Program was carried out at Charters Towers Central Primary School, with 200 students growing and releasing *Wolbachia* carrying mosquitoes
- Douglas Shire - community mosquito releases were carried out in cooperation with the Douglas Shire Council (24 staff constituting 20% of the total workforce signed up to receive a Mozzie Box (mosquito egg release kit) once a fortnight for 8 weeks; Rotary Club of Mossman distributed Mozzie Boxes to neighbors and their personal networks
- The Cassowary Coast – the Wolbachia Warriors Program was carried out at Mission Beach State School, with 120 students growing and releasing *Wolbachia* carrying mosquitoes

**Figure 16.**
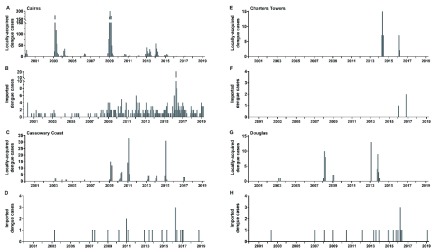
Dengue case notifications per month, January 2000 – March 2019, in four local government areas where *Wolbachia* mosquitoes have been released. Notifications include laboratory-confirmed and probable dengue cases, classified as locally-acquired ( **A**, **C**, **E**, **G**) or imported ( **B**, **D**, **F**, **H**) based on a history of overseas travel to a dengue-affected country during the period 3 – 12 days prior to illness onset. Case location was determined from geolocated address information from the Cairns and Townsville public health unit operational databases, where available, otherwise from suburb in the NoCS case record.

Over the past 20 years the prevalence of *Ae. aegypti* mosquitoes, coupled with viremic international travelers has resulted in episodic local dengue outbreaks in northern Queensland. Between January 2000 and March 2019, 2,086 locally-acquired cases and 301 imported cases (travel history not documented for 3 cases) were notified to the Queensland Health notifiable conditions system from across the Cairns, Cassowary Coast, Charters Towers and Douglas local government areas (LGAs) ( Figure 16). Nearly all locally-acquired cases (94%), and two-thirds of imported cases, were notified during the monsoonal months December – May, and the large majority (87%) were in the populous Cairns regional area. The Department of Health responded in 1998 with emergency vector control activities through a specialized unit (Dengue Action Response Team) to conduct extensive source reduction and chemical intervention activities that included targeted interior residual spray, deployment of lethal ovitraps, and application of larvicides to water holding containers ( Queensland Health, 2015). Despite these efforts, and with the increasing numbers of imported dengue cases every year from 2000–2019 ( Figure 16B), local dengue transmission occurred most years in Cairns ( Figure 16A), with large outbreaks in 2003 (450 local cases) and 2009 (776 local cases).

**Figure 17.**
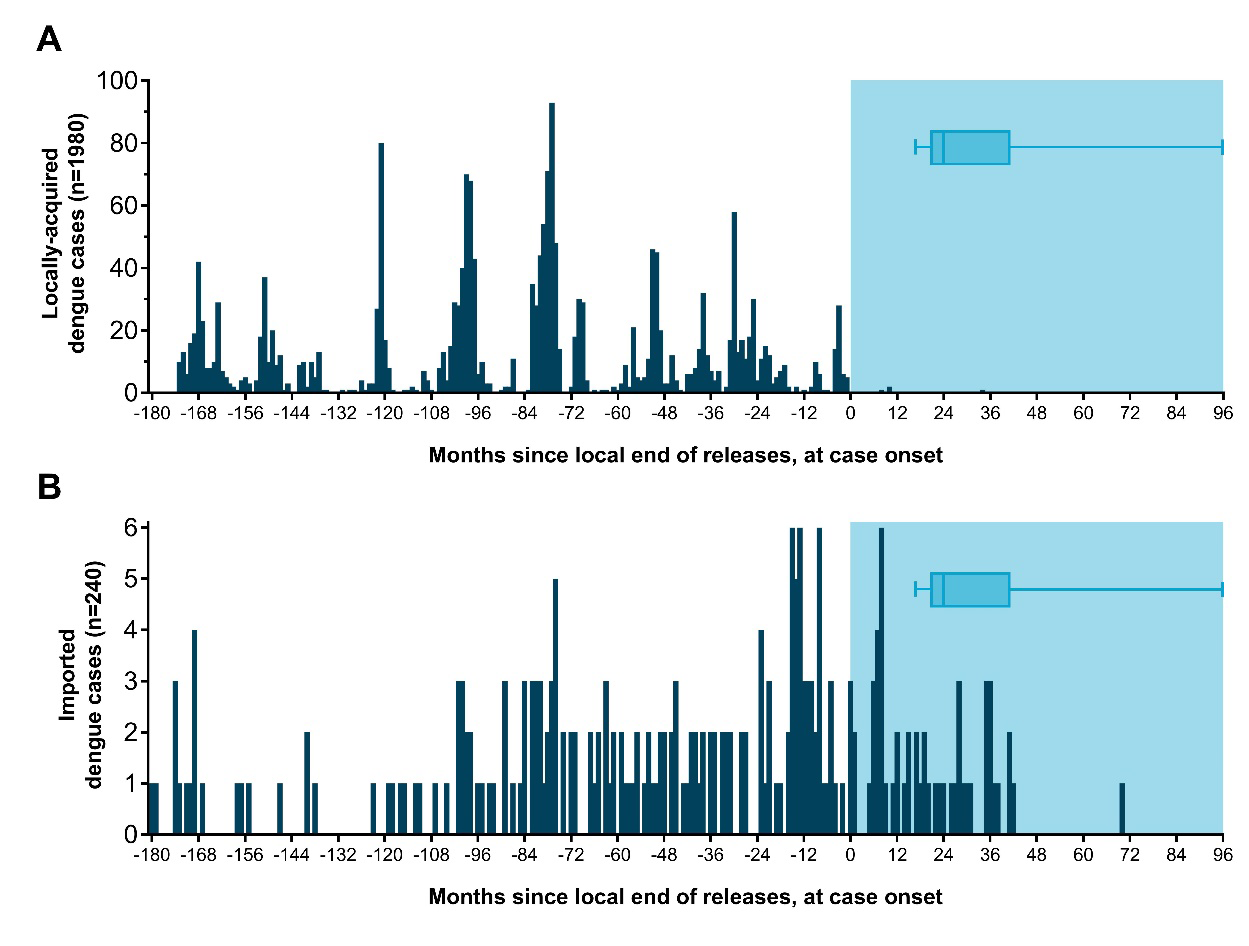
Timing of dengue case notifications January 2000 – March 2019 from *Wolbachia* intervention areas, relative to *Wolbachia* deployments. The date of case onset is scaled relative to the date that local *Wolbachia* releases were completed or, for the five central Cairns non-release areas where *Wolbachia* established, the inferred date when local *Wolbachia* frequency reached 80%. In the post-intervention period (blue shaded area), imported cases continue to occur ( **B**) but locally-acquired cases have been effectively eliminated ( **A**). The post-intervention case surveillance period is variable across the release areas, due to staggered releases from Jan 2011 to May 2017: the median post-intervention observation period is 24 months (IQR 21–41 months, range 17–96 months), as shown in the box plots. The x-axis is left-censored at 15 years pre-release (excludes 8 local cases and 5 imported cases occurring >15 years pre-release).

The staggered deployment of *Wolbachia* across Cairns in 2011 – 2017, and into the urban centres of the Cassowary Coast, Charters Towers and Douglas regions in 2016 and 2017, led to *Wolbachia* establishment throughout communities with a total resident population of 165,000 people. When the timing of notified dengue cases is scaled relative to the local completion of *Wolbachia* deployments ( Figure 17), this demonstrates the near elimination of locally-acquired dengue cases from *Wolbachia*-treated communities. Only four local cases were notified from *Wolbachia*-treated areas in the eight years since completion of the first releases in March 2011, while dengue case importations continued in these areas. All four of these local cases were notified in January – March 2014, more than five years ago, and three of the four were from early central Cairns release areas (Parramatta Park and Westcourt) which were at that time 8-10 months post-release and surrounded by untreated areas.

**Figure 18.**
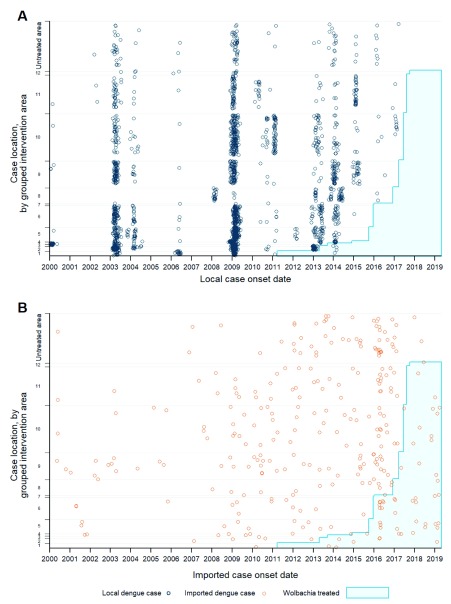
Notifications of locally-acquired ( **A**) and imported ( **B**) dengue cases relative to *Wolbachia* deployments, in Cairns, Cassowary Coast, Charters Towers and Douglas local government areas, January 2000 – March 2019. Cases are plotted by date of illness onset, and by grouped intervention area determined from geolocated address, or from suburb where address was unavailable. Intervention areas were grouped by the calendar quarter in which releases were completed or, for the five central Cairns non-release areas where *Wolbachia* established, the inferred date when local *Wolbachia* frequency reached 80%. Cases located in the four LGAs, but outside of any *Wolbachia* established area, are shown in ‘Untreated area’ at the top of each graph. The Y-axis scale is proportionate to the population size of each intervention area (or untreated area). The grouped intervention areas, and the quarter in which they were considered *Wolbachia*-treated for epidemiological purposes, were as follows: Group 1: Q1 2011 (GV, YK); Group 2: Q2 2013 (EHW, PP, WC); Group 3: Q3 2013 (BA, MB); Group 4: Q4 2014 (BU1, BU3, CN1, SF1-3); Group 5: Q3 2015 (MDA, MRA, BUN NR, EA); Group 6: Q4 2015 (M BU2, BUX1, BUX2, MDA NR1, MDA NR2, MS, OO, WCX1, WCX2, WO, WR); Group 7: Q1 2016 (WC NR); Group 8: Q4 2016 (CB, CT, MG, MN, MO, PD); Group 9: Q1 2017 (BP, CNX, EDM); Group 10: Q2 2017 (BEL, BH, BRN, BUX3, EHX, FFP, IAE, IES, INN, KB, MOU, MRAX, MSX, MUN, POR, PPX, SJO, WAN, WFX, WRX); Group 11: Q3 2017 (AER, BBY, CB, ELA, HB, FW, KWB, NMB, PC, SMB, SMF, TRB, TRP, TUL, WGB); Group 12: Q4 2017 (PE).

The spatial distribution of locally-acquired and imported dengue cases across the intervention areas is illustrated in Figure 18, which highlights that the 2003 and 2009 outbreaks widely affected most parts of Cairns and the other urban centers. In striking contrast, in the years following the first *Wolbachia* releases in Cairns in 2011, substantial local transmission continued to occur but was concentrated each season within the ever-diminishing area in which *Wolbachia* had not yet been released. In total, 515 locally-acquired dengue cases were notified across the four regions since 2011, of which only four have been located in *Wolbachia*-treated areas.

In an interrupted time series analysis of this case notification data, the regression model estimate of *Wolbachia* intervention effect indicated a 96% reduction in dengue incidence in *Wolbachia* treated populations (95% confidence interval: 84 – 99%), adjusted for season, imported cases, and allowing for temporal autocorrelation of cases.

The *w*Mel strain of *Wolbachia* has been deployed across the major regional cites of Cairns and Townsville, as well as nearby smaller regional communities that have historically been affected by dengue transmission. Consistent with modelling projections ( Ferguson *et al.*, 2015), *Wolbachia* deployments have been associated with cessation of local dengue transmission. Alternative explanations for the absence of local dengue transmission are unlikely; there has been no change to local vector control activities and the number of notified imported dengue cases has not diminished with time. Ongoing long-term monitoring is expected to confirm the durability of *Wolbachia* and its persistence in local *Ae. aegypti* populations ( Ritchie *et al.*, 2018), and the “dengue proofed” status of northern Queensland. Additional public health evidence for *Wolbachia*’s impact on dengue transmission will be delivered by a large cluster randomized trial currently underway in Indonesia (Clinical Trials.gov Identifier: NCT03055585).

This current report demonstrates *Wolbachia* deployment is scalable, safe, long-lasting, acceptable to communities and is associated with cessation of dengue transmission. With the global burden of dengue clearly not adequately controlled by existing public health tools, the *Wolbachia* approach should be considered for communities at risk of or endemic for dengue.

## Data availability

### Underlying data

Figshare: CNS_Monitoring_Results.xlsx. https://doi.org/10.6084/m9.figshare.9831113 ( Ryan, 2019).

This project contains all data underlying results presented in Figure 6– Figure 15.

Human dengue case notification data was provided to us by Queensland Health. The conditions of the release of the raw dengue case notifications data to us by the Communicable Disease Branch of Queensland Health do not permit further sharing to a third party. This data (locally acquired and imported dengue case notifications) can be acquired by application to Queensland Health: https://www.health.qld.gov.au/clinical-practice/guidelines-procedures/diseases-infection/surveillance/reports/notifiable/data-request. Access to data will be considered following completion of the Data request form (PDF, 237 kB).

Data deposited with Figshare are available under the terms of the Creative Commons Attribution 4.0 International license (CC-BY 4.0).
