# Supplementary material for: Establishment of wMel Wolbachia in Aedes aegypti mosquitoes and reduction of local dengue transmission in Cairns and surrounding locations in northern Queensland, Australia
Source: Gates Open Res. 2020 Apr 8;3:1547. Originally published 2019 Sep 26. [Version 2] doi: 10.12688/gatesopenres.13061.2 (PMC6801363; doi:10.12688/gatesopenres.13061.2)
Supplement: Peer review history

# Peer review: Reviewer response for version 1

**DOI:** 10.21956/gatesopenres.14191.r28016

**Published:** 2019-10-18

**Authors:** Luca Facchinelli

**Affiliations:** 1 Department of Vector Biology, Liverpool School of Tropical Medicine, Liverpool, UK

This is a well-conceived and clearly written manuscript that gives a whole picture of the results obtained in dengue control by using *Wolbachia*-infected mosquitoes in Australia.

 As stated in the manuscript, this work was needed to summarise the methodology and the results from several studies and interventions carried out in Queensland. It describes the steps undertaken to achieve *Aedes aegypti* population replacement, from the community and stakeholder engagement to the mathematical modelling and the strategies to release *Wolbachia*-infected mosquitoes.

 The conclusions are fully justified and the reduction in dengue incidence achieved by spreading *Wolbachia* into the local *Ae. aegypti* populations is unprecedented worldwide. No other tool has shown such a consistent and egalitarian result in fighting dengue virus transmission so far.

 In my opinion there will be two crucial tests for the *Wolbachia* technology. The first one will be in highly urbanised areas of LMIC where arbovirus transmission is high and both *Ae. aegypti* and *Aedes albopictus* thrive and live in sympatry. The second one is time related: being *Wolbachia*- *Ae. aegypti *is a very recent symbiosis, will we continue observing the same arbovirus blocking effects in the decades ahead?

 As I mentioned above, the manuscript is clearly written, and I only have a minor comment below:

 The lower than expected spreading of *Wolbachia* in the *Ae. aegypti *population across the Pyramid Estate area is extensively commented in the Discussion section (Page 20-22), but I suggest citing Schmidt *et al.* (2018 ^1^), and comment on their findings to be thorough on this topic and better support pilot studies using *Wolbachia*-infected *Ae. aegypti* in different ecological settings.

 Hope this is helpful.

I confirm that I have read this submission and believe that I have an appropriate level of expertise to confirm that it is of an acceptable scientific standard.

## References

[1] : Fine-scale landscape genomics helps explain the slow spatial spread of Wolbachia through the Aedes aegypti population in Cairns, Australia. *Heredity (Edinb)*.120(5) : 10.1038/s41437-017-0039-9 386-395 10.1038/s41437-017-0039-9 29358725PMC5889405

# Response

**Published:** 2020-01-21

**Authors:** Peter Ryan

**Affiliations:** World Mosquito Program, Vietnam

Response to Review 2:

 1. The reference to Schmidt et al. 2018 and the text below has been added to page 20.

 "... and this was possibly due to barriers to *Ae. aegypti* dispersal, higher incidence of long-range *Ae. aegypti* dispersal, and intergenerational loss of *Wolbachia *(Schmidt *et al.*, 2018)."

# Peer review: Reviewer response for version 1

**DOI:** 10.21956/gatesopenres.14191.r27997

**Published:** 2019-10-08

**Authors:** Jeffrey R. Powell

**Affiliations:** 1 Yale University, New Haven, CT, USA

This is an impressive document summarizing a massive amount of work and data collection. It is very timely and helpful in assessing the efficacy of the Wolbachia release work that has been going on for about ten years. It pulls together a scattered literature distilling the essence of previous publications. So overall, I have no reservations about recommending this to be indexed. 

 However, lest the authors think I’m brain dead, I do have some comments:

1. It would have been useful to have some final summing up of what was learned, especially with regard to strategies to establish Wolbachia. Are egg or adult releases more effective? Why were some areas more difficult to get established than others? Is this due to lack of reaching the crucial threshold or other factors? Is season important? 
2. Figures could be clearer. For example, putting on the graphs the city or region would be useful, e.g. Figure 6 might have Cairns in the graph rather than relying on the reader to remember all the acronyms. Labeling Figure 8 as “Egg Releases”. It is annoying to need to read through the dense captions to get the gist of the information.
3. Seems an annoying nuisance to have allowed releases of wAlbB males released for a while in some areas. Did this affect population sizes? 
4. In many of the graphs, the y-axis is simply %. Sample size would be interesting to know. BGS traps are not particularly efficient and I suspect some sample sizes were pretty small.
5. A bit more about what was learned about the spread would be useful. Is the 100-200m/year still the case everywhere? It is concluded on page 22 that releases “do not need to be undertaken in all areas”, but it is not clear what this really means.

I confirm that I have read this submission and believe that I have an appropriate level of expertise to confirm that it is of an acceptable scientific standard.

# Response

**Published:** 2020-01-21

**Authors:** Peter Ryan

**Affiliations:** World Mosquito Program, Vietnam

**Response to Reviewer 1:**

 1. The following has been added to the Results and Discussion section:

 "Overall, short-term releases of between 5-23 weeks involving either egg or adult stages, resulted in the establishment of *Wolbachia* in mosquito populations across all release areas. There were no clear differences between egg or adult releases in terms of *Wolbachia* establishment. The overall duration of egg and adult release periods were similar (average 12 weeks duration for both egg and adult releases), although egg releases were generally undertaken every 2 weeks compared with weekly adult mosquito releases. Although *Ae. aegypti* populations varied seasonally across the study areas, there were no clear seasonal effects on *Wolbachia* establishment, indicating that under the north Queensland conditions releases can be undertaken year round. Operationally, egg releases provided advantages over adult mosquito releases in that there was no need to rear immatures stages to adults in a local insectary. For egg releases, eggs were produced centrally in an insectary, and then transferred to the field and placed into mosquito release containers, either by staff or via community members themselves. These simple, low-cost egg release methods may represent a more scalable approach for future large-scale implementations, particularly in low resource settings where infrastructure for mass rearing of adult mosquitoes is limited."

 2. Heading has been added to each figure indicating location and release type.

 3. *w*AlbB releases were undertaken as part of an independent project. Our sampling strategy using BG traps was not designed to assess the *Ae. aegypti* population size. While we detected *w*AlbB *Ae. aegypti* in some BG traps, the geographic extent of the *w*AlbB releases was unknown.

 4. The numbers of mosquitoes collected from BG traps and screened for *Wolbachia* varied by location and by season. As each frequency graph had up to six different release locations and individual locations had between 23-145 frequency calculations, we were unable to show the sample size for each calculation. The raw data, including sample sizes, for every location and frequency calculation, were listed in the underlying data (CNS_Montoring_Results.xlsx). Overall mean sample size was 32.4 *Ae. aegypti* per collection period (median 16.0, interquartile range 6.0 - 38.0).

 5. The monitoring in the five non-release areas did not specifically measure wave speed as was the aim in Schmidt *et al*., 2017. The non-release areas in the current study were either small areas (0.13 - 0.53 km ^2^) surrounded by areas where *Wolbachia* had been released, or an isolated area (1.99 km ^2^) separated by a main highway from a *Wolbachia* release site. The spread and establishment of *Wolbachia* in all of these areas is consistent with previous estimates of *Wolbachia* spread at 100-200m per year as previously defined by Schmidt *et al.,* 2017. As we point out in the discussion, the main implication of this is the potential for more efficient deployment strategies where "areas are left intentionally with no releases and instead rely on natural spreading of *Wolbachia*."
